# GPX4 is a key ferroptosis regulator orchestrating T cells and CAR-T-cells sensitivity to ferroptosis

**DOI:** 10.1007/s00262-025-04133-w

**Published:** 2025-08-04

**Authors:** Marta Kłopotowska, Iwona Baranowska, Szymon Hajduk, Anna Jurga, Natalia Leśniowska, Michał Łaźniewski, Monika Granica, Marta Krawczyk, Milena Dziewicka, Agnieszka Graczyk, Jan Słupski, Radosław Zagożdżon, Dariusz Plewczynski, Magdalena Winiarska, Malgorzata Bajor

**Affiliations:** 1 Department of Immunology, Mossakowski Medical Research Institute Polish Academy of Sciences, 5 Adolfa Pawinskiego St., 02-106 Warsaw, Poland; 2 Immunooncology Students’ Science Association, Medical University of Warsaw, Warsaw, Poland; 3 Department of Bacteriology and Biocontamination Control, National Institute of Public Health NIH—National Research Institute, 24 Chocimska St., 00-791 Warsaw, Poland; 4 Centre for Advanced Materials and Technologies, Warsaw University of Technology, 19 Poleczki St., 02-822 Warsaw, Poland; 5 Department of Immunology, Medical University of Warsaw, 5 Jana Nielubowicza St., 02-097 Warsaw, Poland; 6 Doctoral School, Medical University of Warsaw, 81 Zwirki i Wigury St., 02-091 Warsaw, Poland; 7 Doctoral School of Translational Medicine, Mossakowski Medical Research Institute, Polish Academy of Sciences, 5 Adolfa Pawinskiego St., 02-106 Warsaw, Poland; 8 Laboratory of Cellular and Genetic Therapies, Medical University of Warsaw, 02-097 Warsaw, Poland; 9 Centre of New Technologies, University of Warsaw, 2C Banacha St., 02-097 Warsaw, Poland; 10 Faculty of Mathematics and Information Science, Warsaw University of Technology, 75 Koszykowa St., 00-662 Warsaw, Poland

**Keywords:** Ferroptosis, T cells, Chimeric antigen receptor, GPX4, Lipid metabolism, Immunotherapy

## Abstract

Induction of ferroptosis, an iron-dependent form of regulated cell death, holds promise as a strategy to overcome tumor resistance to conventional therapies and enhance immunotherapy responses. However, while the susceptibility of tumor cells to ferroptosis is extensively studied, limited data exists on the vulnerability of immune cells to disturbed iron balance and lipid peroxidation. Here, we found that T-cell stimulation rewires iron and redox homeostasis and by increasing levels of reactive oxygen species and labile iron promotes lipid peroxidation and T-cells’ ferroptosis. Upon stimulation, we detected changes in the balance of ferroptosis-suppressive proteins, including decrease of GPX4. Subsequently, we identified GPX4 as a master regulator orchestrating T/CAR-T-cells’ sensitivity to ferroptosis and observed that GPX4 inhibitors impair CAR-T cells’ antitumor functions. Our study demonstrated differential GPX4 expression and diverse susceptibility to ferroptosis between CD4⁺ and CD8⁺ T cells. Among analyzed subsets of naïve, central memory (CM), effector memory (EM), and terminally differentiated effector memory (TEMRA), CD8⁺ EM and CD8⁺ TEMRA cells exhibited the highest sensitivity to ferroptosis. We also showed that ferroptosis limited the anti-tumor efficacy of CAR-T cells, while ferroptosis inhibition improved their therapeutic effect, both in vitro and in vivo. Our findings are not only important to understand vulnerabilities of CAR-T cells but may also hold particular significance for their therapeutic development. In this context, future anticancer therapies should be carefully designed to selectively induce the ferroptosis of tumor cells without impeding cytotoxic cells’ antitumor efficacy. Additionally, we postulate that promoting less differentiated phenotype of CAR-T cells should be exploited therapeutically to create CAR-T products characterized by decreased sensitivity to ferroptosis within tumor microenvironment.

**Supplementary Information:**

The online version contains supplementary material available at 10.1007/s00262-025-04133-w.

## Introduction

Ferroptosis has gathered increased attention as a promising anticancer strategy [1–4]. It has been shown that manipulating lipid metabolism or redox balance can induce ferroptosis in various cancer types, including both solid tumors and hematological malignancies [5–8].

Ferroptosis is a free radical-mediated process that results in iron-dependent excessive oxidation and subsequent degradation of lipids containing polyunsaturated fatty acids (PUFA) [9]. In addition to an increased peroxidation of lipids in the cell membrane and an accumulation of labile iron pool inside the cell, ferroptosis is characterized by changes in the redox state, leading to increased production of reactive oxygen species (ROS) and a concomitant impairment of the cell’s antioxidant defense systems [10]. To prevent ferroptosis, cells activate key defense systems such as cyst(e)ine–glutathione (GSH)–glutathione peroxidase 4 (GPX4) and ferroptosis suppressor protein 1 (FSP1)/coenzyme Q10 (CoQ10) [11].

While ferroptosis of tumor cells is widely studied, the effects of ferroptosis on immune cells, particularly in the tumor microenvironment (TME), still remain poorly understood. Recent evidence suggests that neutrophils within tumors can undergo spontaneous ferroptosis, thus suppressing T cell activity [12]. Conversely, Zhao et al. have demonstrated the upregulation of ferroptosis-related genes in neutrophils found in metastatic sites, potentially indicating their defensive mechanism against ferroptosis [13]. Notably, T cells are more prone to ferroptosis than neutrophils, as evidenced by lipidomic studies[14] 

Furthermore, in the TME, cystine consumption by tumor cells disrupts the cystine/glutamate exchange in CD8^+^ T cells, leading to increased CD36 expression, fatty acids uptake, lipid accumulation, abnormal ROS production, and ultimately T cell exhaustion and ferroptosis. [15–17] Consistently, blocking CD36-induced ferroptosis, in combination with immune checkpoint inhibitors, has been shown to enhance CD8^+^ T cell anticancer activity. [16] On the other hand, CD8^+^ T cells have been shown to promote ferroptosis in tumor cells by secreting interferon-γ (IFNγ) [18]. To mount the effective antitumor response, T cells first undergo stimulation before interacting with cancer cells within the tumor niche [19]. Therefore, in this study we investigated how T cells’ stimulation affects their susceptibility to ferroptosis. We demonstrated that T cells’ stimulation significantly decreases GPX4 expression and affects the balance between processes promoting and protecting against ferroptosis. We also observed that CAR-T cells are susceptible to ferroptosis induced by GPX4 inhibition that reduces their antitumor potential. Chimeric antigen receptor (CAR)-engineered T-cell therapies, although very effective in hematological malignancies, still faces several obstacles in eliminating solid tumors. Limited responses from exhausted and senescent CAR-T cells may be attributed, among others, to inadequate culture/manufacture conditions. Recently, substantial advancements have been made in optimizing CAR-T culture conditions and time. It has been shown that short-manufactured CAR T cells exhibited more robust tumor control compared to long-manufactured ones [20]. Moreover, it has been reported that CAR-T cells treated with various cytokines display different phenotype and persistence in vivo. Accordingly, CAR-T cells treated with IL-21 exhibited characteristics indicative of delayed senescence, reduced glycolytic activity and reduced sensitivity to ferroptosis [21]. Another reason for impaired CAR-T efficacy in solid tumors is the hostile, immunosuppressive tumor microenvironment (TME), characterized by chronic oxidative stress. This stress contributes to mitochondrial dysfunction, lipid peroxidation, and impaired antioxidant defense systems, all of which are metabolic features characteristic of ferroptosis. In this context, we also show that inhibition of ferroptosis with liproxstatin 1 (Lip-1) protects CAR-T cells and improves the effective eradication of cancer cells, both in vitro and in vivo.

## Materials and methods

### Cell lines

Raji and MCF7 cell lines were purchased from ATCC or the European Collection of Cell Cultures (Wiltshire, UK). The cells were cultured in RPMI-1640 medium (Sigma-Aldrich, St Louis, MO, USA) supplemented with 10% fetal bovine serum (FBS) (Sigma-Aldrich), 2 mM L-glutamine (Sigma-Aldrich) and 1% antibiotic–penicillin/streptomycin (Sigma-Aldrich) (referred as full RPMI medium) in a humidified atmosphere containing 5% CO_2_. All the cell lines were maintained through continuous passaging and were confirmed to be free of contamination with Mycoplasma spp. For luciferase-based assays cell lines (Raji and MCF7) were modified with plasmid pLenti7.3/V5 TOPO-RedLuc encoding the red luciferase gene and green fluorescent protein, as described previously. [22]

### Reagents

All tested compounds GPX4 inhibitors: RSL3 (Selleckchem, S8155) and ML162 (Merck, SML2561), iFSP1 (Selleckchem, S9663), liproxstatin-1 (Merck, SML1414), liproxstatin-1 for in vivo experiments (Chem-Norm, TBW04068), ferrostatin-1 (Merck, SML0583), Z-VAD-FMK (Selleckchem, S7023), necrostatin-1 (Sigma, N9037) were dissolved in DMSO to obtain 10 mM stock solutions and kept at -20 or -80 °C according to manufacturer’s recommendations. Further dilutions were performed in culture medium, directly before each experiment. Deferoxamine (Desferal, Novartis leftovers donated by patients) was dissolved in dH_2_O and was kept at -20 °C.

### T cell isolation and stimulation

Human primary T cells were isolated from buffy coats of healthy donors obtained from the Regional Blood Center in Warsaw, Poland, the procedure was approved by the Local Bioethics Committee (approval number: AKBE/62/2021) and was performed in accordance with the Declaration of Helsinki. Initially, mononuclear cells were isolated by density gradient centrifugation using Lymphoprep (STEMCELL Technologies Canada, Inc.). Subsequently, T cells were magnetically separated from mononuclear cells with negative selection using EasySep™ Human T Cell Isolation Kit (STEMCELL Technologies Canada, Inc.). T cells were stimulated with the magnetic beads coated with monoclonal antibodies against CD3 and CD28 molecules (Dynabeads Human T-Activator CD3/CD28, Thermo Fisher Scientific) (bead to cell ratio 1:5) and were cultured in full RPMI medium supplemented with 100 U/mL of IL-2 (Peprotech). After 5 days the beads were removed and T cells were further cultured in the presence of IL-2 only. Study adheres to the Declaration of Helsinki.

### T cell viability assays

Unstimulated T cells, stimulated T cells or CAR-T cells were treated with tested compounds for an indicated time, 24 or 48 h. Subsequently, the viability of the cells was assessed by propidium iodide (PI, 1 μg/mL, Sigma-Aldrich) staining, followed by flow cytometry analysis BD FACSCantoII and HTS sampler.

### T cell proliferation assay

Human primary T cells were resuspended in PBS (1 × 10^6^/ml) and stained with Cell Trace Violet (CTV) dye (Thermo Fisher Scientific) for 20 min at 37 °C at a final CTV concentration of 2.5 µM. Subsequently, T cells were washed and seeded onto a 96-round-bottom plate (2 × 10^4^ cells per well) in full RPMI medium in the presence of IL-2 (100 U/mL), Dynabeads Human T-Activator CD3/CD28 (beads to cell ratio 2:1) and increasing concentrations of RSL3 with or without Lip-1. After 3 and 6 days of incubation, the T cells were stained with DRAQ7 viability stain (BioLegend) and analyzed on BD FACSCantoII and HTS sampler.

### T cell subsets evaluation after RSL3 treatment

Stimulated T cells were seeded with RSL3 for 24 h. Following treatment, the lowest effective concentrations were selected for subsequent phenotypic analysis. Briefly, cells were washed, stained with BD Horizon™Fixable Viability Stain 510 (BD Biosciences) to exclude dead cells form further analysis. Subsequently, T cells were stained with antibodies against CD4, CD8, CCR7 and CD45RA. The proportions of specific T cell subsets were then analyzed by flow cytometry.

### BD Lyoplate™ human cell surface marker screening panel

To screen the expression of surface markers on unstimulated and stimulated T cells, we used the BD Lyoplate™ Human Cell Surface Marker Screening Panel (BD Biosciences). Briefly, T cells at different days after stimulation were washed and resuspended in staining buffer (PBS supplemented with 2% FBS and 1 mM EDTA). For a single staining 1 × 10^5^ T cells were used. Staining was performed according to the manufacturer’s recommendations, with minor modifications. After staining the cells were fixed with BD Cytofix (BD Biosciences) solution and stored for acquisition up to 24 h in the dark at 4 °C.

### Flow cytometry staining

Extracellular staining for surface antigen was performed in a staining buffer (PBS supplemented with 1 mM EDTA and 2% FBS) for 20 min at room temperature onto a 96-round-bottom plate. For intracellular staining, the cells were fixed with BD Cytofix buffer (BD Biosciences), permeabilized with BD Perm/Wash buffer and stained at 4 °C for 30 min. Next, cells were washed in Perm/Wash buffer, resuspended in staining buffer and analyzed with BD FACSCantoII (BD Biosciences) and HTS sampler. Antibodies used for staining are listed in Table 1. All flow cytometry data were analyzed using FlowJo (Becton Dickinson).

**Table 1:** Flow cytometry antibodies

Antibody	Fluorochrome	Catalog number	Company
CD71 (transferrin receptor)	APC	17-0719-42	Invitrogen
Ferroportin/SLC40A1	PE	NBP1-21502PE	Novus Biologicals
CD340 (erbB2/HER-2)	APC	324,408	BioLegend
goat anti-human IgG, Fcγ fragment specific	AF647	109-606-098	Jackson ImmunoResearch Labs
CD45RA	FITC	11-0458-42	eBioscience
CCR7	BV421	404-1979-42	eBioscience
CD8	APC	17-0088-42	eBioscience
CD4	PE-Cy7	557852	BD Biosciences

### Lipid peroxidation assay

Lipid peroxidation was evaluated in T cells either at a steady state or following RSL3 treatment using the fluorescent lipid peroxidation sensor named BODIPY 581/591 C11 (ThermoFisher, D3861). Briefly, T cells were seeded onto a 96-round-bottom plate at cell density 1 × 10^6^/ml and incubated with increasing concentrations of RSL3 in the presence or absence of Lip-1 for 20 h. The next day, T cells were centrifuged and resuspended in full RPMI medium containing 0.5 µM BODIPY 581/591 C11 reagent (100 µl/well) and incubated at 37 °C for 30 min. Subsequently, the cells were washed three times and analyzed on BD FACSCantoII and HTS sampler. The level of lipid ROS was assessed as an increase in oxidized C11-BODIPY (green fluorescence).

#### Labile iron pool detection

Intracellular Fe^2+^ level was evaluated with the fluorescent probe FerroOrange (Dojindo). The cells were seeded onto a 96-round-bottom plate at cell number 2 × 10^5^/well, washed three times with PBS, and then resuspended in HBSS buffer. In experiments with deferoxamine pretreatment, the cells were preincubated with 1 mM or 2 mM deferoxamine and incubated for 30 min at 37 °C. Subsequently, 2 times concentrated FerroOrange probe was added to the wells at a final concentration of 1 µM and incubated for 30 min at 37 °C. After incubation, cells were analyzed without washing on a BD LSRFortessa X20 instrument (BD Biosciences) and PE channel.

#### Intracellular ROS detection

Intracellular ROS were determined with fluorescent probes CellROX Deep Red and CellROX Green (Thermo Fisher Scientific). Briefly, T cells were seeded onto a 96-round-bottom plate at the density of 5 × 10^5^ cells/mL and incubated with the CellROX Deep Red or CellROX Green reagent at 37 °C, 5% CO_2_ for 30 min. After washing, the cells were analyzed on a BD FACSCantoII flow cytometer (BD Biosciences).

#### GSH detection

To determine GSH level in T cells the Intracellular glutathione (GSH) Detection Assay Kit (Abcam, ab112132) was applied. Briefly, T cells were seeded onto a 96-round-bottom plate at cell density 1 × 10^6^/ml and incubated with Thiol Green fluorescent probe (diluted 1:10,000) for 30 min at 37 °C. After incubation, the cells were washed and analyzed using flow cytometry and green fluorescence.

#### CAR constructs and lentiviral T cell modification

In this study, we utilized two CD19 CAR (FMC63 clone) constructs, generously provided by M. Pule from UCL, UK. The first construct includes the CD8 hinge and transmembrane domain, the 41BB costimulatory domain, the CD3ζ signaling domain, and the rituximab recognized-RQR8 epitope for CAR detection. The second CD19 CAR construct consists of the IgG1 half-hinge, the CD28 transmembrane and co-stimulatory domain, and CD3ζ. PD-L1-targeting CAR consists of an atezolizumab-based scFv sequence following an IgG1 half-hinge, CD28 transmembrane region, CD28 costimulatory domain, and CD3ζ signaling domain. HER2 CAR construct consists of a trastuzumab-based scFv sequence following a CD8 hinge and transmembrane domain and a 4-1BB-CD3ζ signaling tail. All constructs were subcloned into the lentiviral pSEW plasmid. T cells were modified with the CAR constructs using a lentiviral transduction system as described previously [23]. The CAR expression on the surface of the T cells was evaluated by flow cytometry 72–96 h after transduction as described in [23]. For transduction, PBMC from healthy donors were stimulated with either PHA-L or anti-CD3 and anti-CD28 soluble antibodies for 48 h, subsequently 1 × 10^6^ cells were used for lentiviral transduction. After the second round of viral spinoculation, cells were stimulated with the magnetic beads coated with monoclonal antibodies against CD3 and CD28 molecules (Dynabeads Human T-Activator CD3/CD28, Thermo Fisher Scientific) (bead to cell ratio 1:1) and were cultured in full RPMI medium supplemented with 100 U/mL of IL-2 (Peprotech). After 7 days the beads were removed and MOCK/CAR T cells were further cultured in the presence of IL-2 (100 U/mL) only up to 5 weeks.

#### RTCA-based killing assay

The HER2 CAR-mediated killing of T cells was monitored with a real-time cell analysis (RTCA) assay. Adherent target MCF7 cells (3 × 10^4^ cells/well) were seeded onto 16-well E-Plate (ACEA Biosciences) in 150 μl of a full RPMI medium. The proliferation of MCF7 cells was monitored in the incubator at 37 °C (5% CO_2_, 95% humidity) for 24 h with the xCELLigence impedance-based RTCA system (ACEA Biosciences). The next day, 100 μl of the medium was aspirated and replaced with the full RPMI medium containing effector cells (control unmodified T cells or HER2 CAR-T cells) at effector to target ratio E:T 2:1. T cells and CAR-T cells were pretreated with increasing concentrations of RSL3 for 5 h and transferred onto target cells without washing out the RSL3-containing medium. The CAR-mediated killing of target cells was monitored for the next 12 h. Analysis was performed using RTCA Software Pro (ACEA Biosciences). The impedance changes (cell index) were normalized to the end value of the target cells’ proliferation and plotted over time as normalized cell index.

#### Luciferase-based cytotoxicity assay

Cell lines previously modified to express the luciferase reporter gene (Red-Luc), were seeded onto the 96-well black plates with a clear bottom (Perkin Elmer) at a cell density of 3 × 10^4^ per well in 100 µl of full RPMI in three or four technical replicates. MCF-7 cells were allowed to adhere for 24 h while suspension Raji cells were directly used in the experimental procedures. For cytotoxicity assays, increasing concentrations of RSL3 were added to the wells and the cells were incubated for 48 h. For luciferase-based killing assays, effector CAR-T cells and control unmodified T cells were added to the wells at different E:T ratios and were cocultured for the next 18 h. For bioluminescence readout Bright-Glo™ Luciferase Assay System (E2610, Promega) was used. The plate was incubated for 5 min in darkness at room temperature and luminescence was measured using Tecan INFINITE M1000 (TECAN).

#### Degranulation and cytokine production assay

Before degranulation and cytokine production assay CAR-T cells were preincubated with RSL3 for 16 h. The next day, tumor cells expressing recognized antigen on the surface were added to appropriate wells at E:T (effector to target) 1:1. CD19 CAR-T cells were incubated with Burkitt’s lymphoma cell line Raji (CD19^+^), HER2 CAR-T cells with breast cancer cell line MCF7 (low HER2^+^) and PD-L1 CAR-T cells with Raji cells, genetically modified to overexpress PD-L1 molecule [23]. Subsequently, Golgi Stop (BD Biosciences, dilution 1:250), Golgi Plug (BD Biosciences, dilution 1:200) and anti-CD107a-PE antibody (BD Biosciences, dilution 1:40) were added and the assay plate was incubated for 4 h at 37 °C and 5% CO_2_. After incubation, the cells were stained with anti-CD3-BV421 antibody and Fixable viability stain 510 (BD Biosciences, dilution 1:200) followed by fixation and permeabilization procedures. Eventually, the cells were stained for cytokines with anti-IFNy-APC (BD Biosciences, dilution 1:100) and anti-TNFα-PECy7 (BD Biosciences, dilution 1:100) antibodies. Degranulation and cytokine production by effector cells was assessed using flow cytometry.

#### Western blotting

For Western blotting, cells were lysed with RIPA lysis buffer (Tris–HCL pH 7.4, NaCl 150 mM, NP-40 1% (v/v), sodium deoxycholate 1% (v/v), SDS 0.1% (v/v)) supplemented with Complete Protease Inhibitor Cocktail and Phosphatase Inhibitor Cocktail (Roche Diagnostics). Protein concentration was measured using the Pierce™ Rapid Gold BCA Protein Assay Kit (Thermo Fisher Scientific) according to the manufacturer’s instructions with minor modifications on the TECAN Infinite M1000 Pro microplate reader. 20 μg of cell lysates were separated in 10, 12 or 15% (v/v) (depending on the molecular weight of detected protein) reducing SDS–polyacrylamide gel. For monitoring protein migration and molecular weight of detected proteins PageRuler™ Prestained Protein Ladder (26617, ThermoFisher Scientific) was used. Subsequently, proteins were transferred onto nitrocellulose membranes and blocked with either 5% (w/v) nonfat milk or 5% Bovine Serum Albumin (w/v) (Kenilworth) in TBST (Tris-buffered saline, pH 7.4 and 0.05% (v/v) Tween-20) and then incubated with the following primary antibodies: anti-GPX4 (Cell Signaling cat. 52455S, dilution 1:1000), anti-FSP1 (Abcam cat. ab302673, dilution 1:1000), anti-ACSL4 (Santa Cruz cat. sc-271800, dilution 1:1000), anti-LOX15 (Abclonal cat. A6864, dilution 1:1000), anti-LC3 (Cell Signaling cat. 4108, dilution 1:1000), and anti-β-actin-HRP (A222, Sigma-Aldrich; dilution 1:50,000. For detection of primary protein bands HRP-conjugated secondary antibodies were used. The blots were exposed to the Super Signal chemiluminescent substrates (Thermo Fisher Scientific). The signal was detected using the ChemiDoc Imaging System (Bio-Rad ChemiDoc MP Imaging System). Uncropped images of all Western blots, as well as the approximate extent of the cropped region, are shown in Suppl. Fig. 7.

#### Animal studies

All in vivo experiments were performed with 8–12-week old male NSG (NOD.Cg-*Prkdc*^*scid*^* Il2rg*^*tm1Wjl*^/SzJ) mice obtained from Charles River Laboratories, which were bred at the Animal Facility of the Mossakowski Medical Research Institute, Polish Academy of Sciences. All experiments were performed in accordance with the guidelines and approved by The Second Local Ethics Committee for the Animal Experimentation, Warsaw University of Life Sciences (number: WAW2/100/2023, WAW2/027/2024). The experiments were carried out in an SPF animal facility with IVC systems. To avoid confounders, all mice were labelled and kept in tagged cages. The cages had an assigned, unchanging place in the rack. Results obtained from individual mice according to the treatment method are presented. The blinding was not applied. The distribution of mice to the experimental groups was random and no animals were excluded during the experiment. The sample size was determined based on the assumed increase in tumor diameter. The experimental group size was calculated by power analysis (for assumed test power 80%) or resource equation approach as described in [24]. The study adheres to ARRIVE guidelines.

#### In vivo experiment

Mice were inoculated subcutaneously with 2 × 10^6^ Raji cells in 50% Matrigel Growth Factor Reduced (Corning) on day 0 of the experiment. Subsequently, on days 4, 7, 10, and 13, 5 × 10^6^ CD19 CAR-T cells were administrated intravenously. Liproxstatin-1 (Chem-Norm) was injected intraperitoneally with 10 mg/kg every day or every other day for 2 consecutive weeks. Control mice received medium or solvent respectively. Tumor growth was monitored three times per week with caliper starting from day 7 of the experiment. Tumor volume was calculated according to the formula volume (mm^3^) = (width^2^ [mm] × length [mm])/2. Mice were sacrificed when the tumor diameter reached 15 mm in at least one dimension. Total number of mice used within this study was 37.

#### RNAseq and bioinformatics analysis

RNAseq analysis was done using two publicly available datasets—GSE59846 [25] and GSE240851 [26]. GSE59846 comprises expression data for two cell types—CD4^+^ naïve and CD4^+^ memory T-cells, each derived from 3 individuals. Each cell type was additionally stimulated for 48 h using beads coated with monoclonal antibodies against the CD3 and CD28. Thus, 12 RNA-seq experiments were carried out; however, due to possible mislabeling of two runs—SRR1531315 and SRR1531316, we excluded them from the downstream analysis. GSE240851 dataset includes transcriptomic data from sorted T cell subsets, specifically CD4⁺ and CD8⁺ T cells, further divided into naïve, central memory (CM), effector memory (EM), and terminally differentiated effector memory (TEMRA) populations, only samples from healthy donors control group were used in the analysis. Raw sequencing data were retrieved from the Sequence Read Archive (SRA; Project ID: PRJNA1005567). Six samples (SRR25643939, SRR25643940, SRR25643942, SRR25644028, SRR25643895, SRR25644030) were excluded from downstream analysis due to a low number of reads mapped to annotated genes. Sample labeling for analyzed datasets is presented in Suppl. Fig. 6. Initially, we assessed library quality with FastQC [27] (v. 0.11.9). Next, trimmomatic [28] (v. 0.39) was used to trim fragments of reads from the 3’ and 5’ ends if their average quality fall below 30. Additionally, reads shorter than 40 bp were excluded at this stage. Processed reads were mapped to the human genome (GRCh38) using the align function available from the Rsubread package [29] (v. 2.12.3). Samtools [30] (v. 1.10) was used to remove unmapped reads. The number of reads associated with human genes, as defined in the Gencode GTF annotation file V40, was calculated using the featureCounts function from the Rsubread package and converted to counts per million (in log2 scale). The differential gene expression analysis was carried out using the limma program [31] (v. 3.54.2). *P*-values were adjusted for multiple tests with the Benjamini–Hochberg procedure. All analyses were carried out in R (v. 4.2.2). Results of the statical test are shown as * for *p*-value ≤ 0.1, **p-value ≤ 0.05; ***, *p*-value < 0.01.

#### Statistical analysis

Statistical analysis was performed with GraphPad Prism 9 (GraphPad Software). To determine data distribution, the Shapiro–Wilk normality test was performed. If data passed the normality test, parametric statistics were used. In all analyses, the tests were two-tailed. For comparison between 2 groups either unpaired or paired t-test was performed, depending on data sets. For the differences between three or more independent groups one-way ANOVA was applied, followed by multiple comparisons tests. For comparison differences between groups with two independent variables, two-way ANOVA with post hoc analysis was performed. All statistically significant differences (*p*-value < 0.05) and their *p*-values were marked on the graphs. Non-significant differences with *p*-value ≥ 0.05 were marked as ns. Data are represented as means with standard deviation. Each dot on the graphs represents the average of 2 technical replicates for a particular donor.

## Results

### T cell stimulation rewires iron and redox homeostasis of T cells

**Fig. 1:**
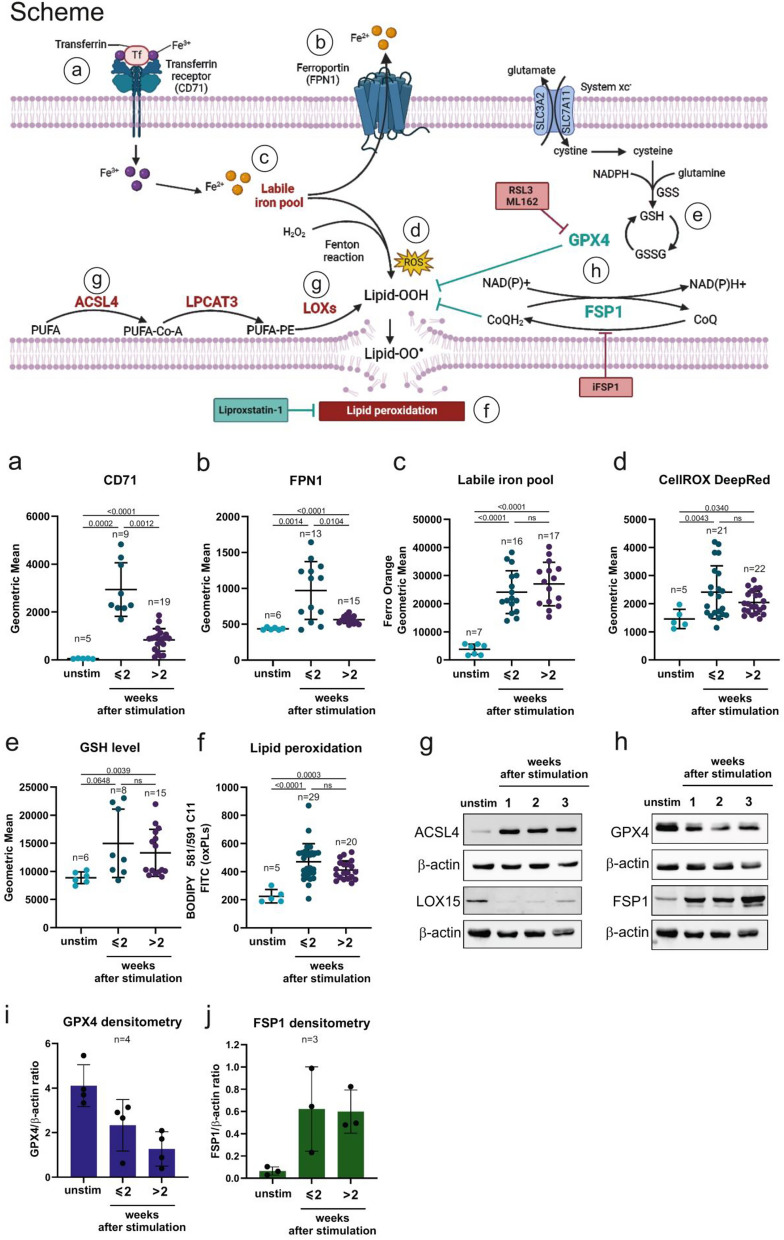
T cells stimulation substantially disturbs lipid, iron, and redox homeostasis Upper part of the figure: Schematic presentation of proteins involved in the ferroptosis pathway. Anti-ferroptotic proteins are marked in green, and pro-ferroptotic proteins are marked in red. Inhibitors used in the experiments are marked in squares. Red square—ferroptosis inducing agents, green squares—ferroptosis inhibitors. Circles with letters a-f indicate ferroptosis-related markers that were analyzed on the graphs below. **a–f.** Analysis of ferroptosis-related markers in unstimulated (unstim) and stimulated T cells cultured for different times after stimulation either up to 2 weeks (≤ 2) or more than 2 weeks (> 2). Data points represent individual donors. Statistical analysis was done with Brown-Forsythe and Welch ANOVA tests with Dunnett; T3 multiple comparisons test, with individual variances computed for each comparison; ns: not significant. All experiments were analyzed using flow cytometry. **a.** Extracellular staining of CD71 (transferrin receptor) on T cells. **b.** Intracellular staining of FPN1 (ferroportin) on T cells. **c.** Labile iron pool in T cells was detected with fluorescent probe FerroOrange. **d.** Intracellular ROS detection using fluorescent probe CellROX DeepRed. **e.** Glutathione level in T cells determined with fluorescent probe Thiol Green. **f.** Lipid peroxidation evaluated with C11-BODIPY 581/591 sensor. **g–h.** Western blotting evaluation of** g**. ACSL4 and LOX15 or **h.** GPX4 and FSP1 protein levels in unstimulated and stimulated T cells at  the following weeks upon stimulation with CD3/CD28 beads and IL-2 (100 U/ml). Data show results from representative donors. β-actin was used as a loading control. **i–j.** Densitometry of GPX4 (i) and FSP1 (j) level in unstimulated and stimulated T cells normalized to β-actin.

To understand the metabolic consequences of T cells’ stimulation, we assessed various regulators of iron and redox homeostasis, as shown in Fig. 1 scheme. For iron homeostasis, we determined the levels of transferrin receptor (CD71) as an iron importer and ferroportin (FPN1), a protein responsible for exporting Fe^2+^ from the cell. Following stimulation, up to two weeks, CD71 protein expression level increased significantly and remained elevated in stimulated T cells compared to unstimulated ones (Fig. 1a). This effect was accompanied by an increase in FPN1 protein level, likely serving as a protective mechanism against toxic iron overload (Fig. 1b). After more than two weeks of  T cells culturing, the level of both CD71 and FPN1 noticeably dropped, but remained elevated as compared with unstimulated T cells. In consequence, following T cell stimulation we observed an increased level of the intracellular labile iron pool (Fig. 1c) that remained elevated even in long-term (over 2 weeks) culture and was only decreased upon deferoxamine treatment, a Fe^2+^ chelator (Suppl. Fig. 1a).

Another observed consequence of T cell stimulation was disturbance in redox homeostasis. To characterize it thoroughly, we assessed ROS levels with the CellROX Deep Red probe, for preferential detection of cytoplasmic and mitochondrial ROS (Fig. 1d), and the CellROX Green reagent (Suppl. Fig. 1b) to detect primarily nuclear-localized ROS. Upon stimulation, we observed an increase in ROS levels in stimulated T cells, although there was no widespread accumulation of ROS. Since ROS are short-lived molecules, the transcriptional activation of antioxidant enzymes can serve as a marker of defense against elevated intracellular ROS levels. Therefore, we reanalyzed RNAseq data in activated CD4^+^ T cells and observed an increased expression, in comparison to unstimulated counterparts, of transcripts encoding antioxidant defense enzymes, including members of the peroxiredoxin and thioredoxin families (Suppl. Fig. 1c). Besides enzymatic antioxidant defense, the increased production of ROS can be also balanced by reduced glutathione (GSH), the most abundant nonprotein thiol in mammalian cells. Therefore, we examined intracellular GSH levels and observed that stimulated T cells exhibited higher GSH levels compared to their unstimulated counterparts (Fig. 1e). We also found that GSH- and glutathione peroxidases (GPXs)-related genes,[32] were up-regulated in stimulated T cells including transcripts for GSS and CHAC1 (Suppl. Fig. 1d). Since GSH synthesis depends on the availability of cysteine that in the cell is derived from the reduction of cellular cystine imported into the cells through the xc- system, a cystine-glutamate exchanger,[33] we subsequently examined the expression of components of the xc- system and found upregulated expression of transcripts for SLC3A2 and SLC7A11 in stimulated CD4^+^ T cells (Suppl. Fig. 1e), altogether supporting the conclusion that stimulation of T cells induces ROS and antioxidant defense systems.

### T cell stimulation promotes lipid peroxidation

Given that ferrous iron (Fe^2+^) and hydrogen peroxide (H_2_O_2_) can react in a process known as the Fenton reaction (Fig. 1 scheme) and generate peroxidized lipids, [10] we subsequently assessed their levels in cell membranes. Upon stimulation, we observed a significant increase in the accumulation of peroxidized lipids in T cell membranes (Fig. 1f) that remained elevated over the prolonged culture, as determined by C11-BODIPY™ 581/591 staining. Since lipid peroxidation is a marker of ferroptotic death, in the next steps we assessed the main pathways involved in the regulation of ferroptosis. The ACSL4–LPCAT3–LOX signaling axis is an intracellular pathway known to promote lipid peroxidation of membrane phospholipids (PLs) containing PUFAs and has been identified as one of the components crucial for ferroptosis execution.[34–36] We examined the expression of ACSL4 and observed its elevation in stimulated T cells both at mRNA (Suppl. Fig. 1f) and protein (Fig. 1g) levels. Also, the mRNA expression for LPCAT3 transcript significantly increased upon T cell stimulation (Suppl. Fig. 1f). Furthermore, in T cells after stimulation we observed a decrease of LOX15 (Fig. 1g), the protein that acts as a ferroptosis-promoting factor by directly oxidizing arachidonoyl (AA) and adrenoyl (AdA) phospholipids (PE) into lipid hydroperoxides.[37] Finally, we examined the expression of two master ferroptosis suppressors, GPX4 and FSP1. In accordance with the accumulation of lipid peroxides upon TCR stimulation, a significant decrease of GPX4, as assessed by Western blotting in various donors, was detected in subsequent weeks after stimulation (Fig. 1h-i, Suppl. Fig. 1g). Surprisingly, FSP1 expression was significantly increased upon stimulation of T cells (Fig. 1h, j, Suppl. Fig. 1g).

In summary, our findings at this step demonstrate that stimulation of T cells substantially disturbs lipid, iron and redox homeostasis that is evidenced by an increased load of labile iron pool, as well as elevated ROS and lipid peroxidation levels. Moreover, T cells upon stimulation downregulate GPX4 and upregulate ACSL4 that are both ferroptosis-promoting effects. At the same time, stimulation of T cells activates the program that confers protection against ferroptosis and includes LOX15 decrease and increase of FSP1. Given that stimulation of T cells changes the balance of ferroptosis-promoting and protecting events, we subsequently employed inhibitors of GPX4, as a primary regulator protecting cells from ferroptosis, to determine T cells’ sensitivity to ferroptosis.

### GPX4 orchestrates T cells’ sensitivity to ferroptosis

**Fig. 2:**
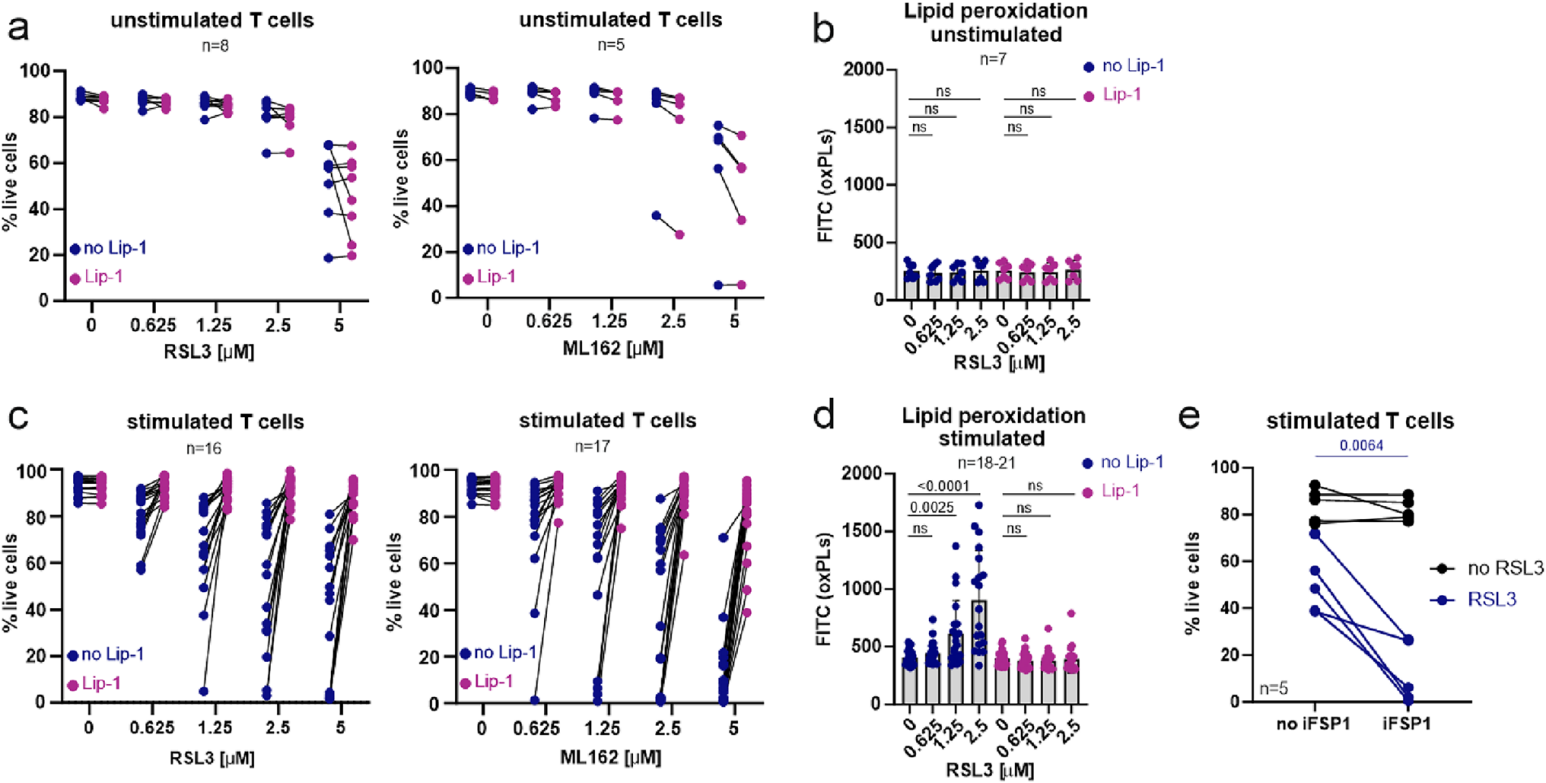
GPX4 inhibition sensitizes T cells and CAR-T cells to ferroptosis. **a.** Sensitivity of unstimulated T cells to GPX4 inhibition. Human primary T cells were isolated from PBMC and incubated with RSL3 or ML162 for 48h ± Lip-1 (0.5 µM). Cell viability was evaluated with flow cytometry and propidium iodide staining. Each data point represents an average of 2 technical replicates for one donor. **b.** Lipid peroxidation of unstimulated T cells upon 24 h incubation with RSL3 evaluated with C11-BODIPY 581/591 staining. Each data point represents averages of 2 technical replicates for one donor. Data are presented as means ± sd. Statistical analysis was done with ordinary two-way ANOVA with Dunnett’s multiple comparisons test; ns: not significant. **c.** Sensitivity of stimulated T cells to GPX4 inhibition. Human primary T cells were stimulated with (CD3/CD28 beads) and IL-2 (100 U/ml). Subsequently, T cells were cultured for at least 2 weeks and were seeded with RSL3 or ML162 for 48 h in the presence or absence of Lip-1 (0.5 µM). Each data point represents an average of 2 technical replicates for one donor. **d.** Lipid peroxidation of stimulated T cells upon 24 h RSL3 treatment evaluated with C11-BODIPY 581/591 staining. Each data point represents averages of 2 technical replicates for one donor. Data are presented as means ± sd. Statistical analysis was done with ordinary two-way ANOVA with Dunnett’s multiple comparisons test; ns: not significant. Statistically significant *p*-values were marked on the graph. **e.** Viability of stimulated T cells incubated with or without iFSP1 (1.25 µM) in combination with RSL3 (2.5–5 µM). The statistic was calculated using a paired t-test

We first observed that unstimulated T cells remained relatively resistant to GPX4 inhibitors (RSL3 or ML162), as demonstrated by their viability in flow cytometry analysis (Fig. 2a) and lipid peroxidation measured by C11-BODIPY™ 581/591 staining (Fig. 2b). Simultaneously, T cells after stimulation acquired sensitivity to GPX4 inhibition in a time-dependent manner. The viability of short-term cultured T cells remained roughly unchanged and was only affected by the highest tested concentrations of GPX4 inhibitors (Suppl. Fig. 2a). This effect was consistent with lack of changes in lipid peroxidation upon GPX inhibitors treatment of T cells in short-term culture (Suppl. Fig. 2b). In contrast, stimulated T cells in long-term culture were sensitive already to the lowest tested concentrations of GPX4 inhibitors (Fig. 2c). Decreased viability of stimulated T cells was further reversible with Lip-1, a ferroptosis-specific inhibitor (Fig. 2c). Consistently with the sensitivity of T cells to ferroptosis, a significant increase in lipid peroxidation reversible by Lip-1, was observed in stimulated T cells, particularly when exposed to higher concentrations of RSL3 (Fig. 2d). In contrast, even if some toxicity of GPX4 inhibitors was observed at the highest doses in unstimulated T cells, it was not reversed by Lip-1 (Fig. 2a). Interestingly, ferroptosis of T cells induced by GPX4 inhibition was further potentiated by iFSP1 (Fig. 2e), an inhibitor that regulates the human FSP1 protein by binding to residue F360 within it. FSP1 is a key component of a nonmitochondrial CoQ antioxidant system that acts in parallel to the canonical glutathione-based GPX4 pathway.[11, 38]

To check whether other types of cell death are induced in T cells by inhibiting GPX4 activity, we employed the pan-caspase apoptosis inhibitor Z-VAD-fmk (Z-VAD), and necroptosis inhibitor necrostatin-1 (Nec-1). They both did not reverse the toxic effects of RSL3 on stimulated T cells (Suppl. Fig. 2d) and simultaneously did not affect the viability of unstimulated T cells (Suppl. Fig. 2c). Moreover, as some reports suggest that the initiation of autophagy could potentially influence the onset of ferroptosis,[39] we performed a Western blot analysis to assess the LC3 protein, which is recognized as an indicator of autophagy. Up to a week following T cell stimulation, we detected increased LC3 protein levels in T cells’ lysates, which was in line with previous research demonstrating that TCR activation triggers autophagy in T cells.[39, 40] However, there was a noticeable decrease in LC3 protein levels in subsequent days of T cell culture, suggesting that autophagy is unlikely to be involved in mediating ferroptosis in this context (Suppl. Fig. 2e). We also checked the influence of GPX4 inhibition on T cells’ proliferation upon stimulation with anti-CD3/CD28 antibodies. After 3 days of stimulation in the presence of increasing concentrations of RSL3, we observed some shifts in the peaks corresponding to the inhibition of the proliferation at the highest RSL3 doses. However, these changes were not reversible by Lip-1 and were further mitigated by day 6 of the culture (Suppl. Fig. 2f). Altogether, our results at this step show that GPX4 inhibition sensitizes T cells to ferroptosis in a manner reversible by Lip-1.

### Distinct T cell subsets are characterized by differential GPX4 expression and varying sensitivity to ferroptosis

**Fig. 3:**
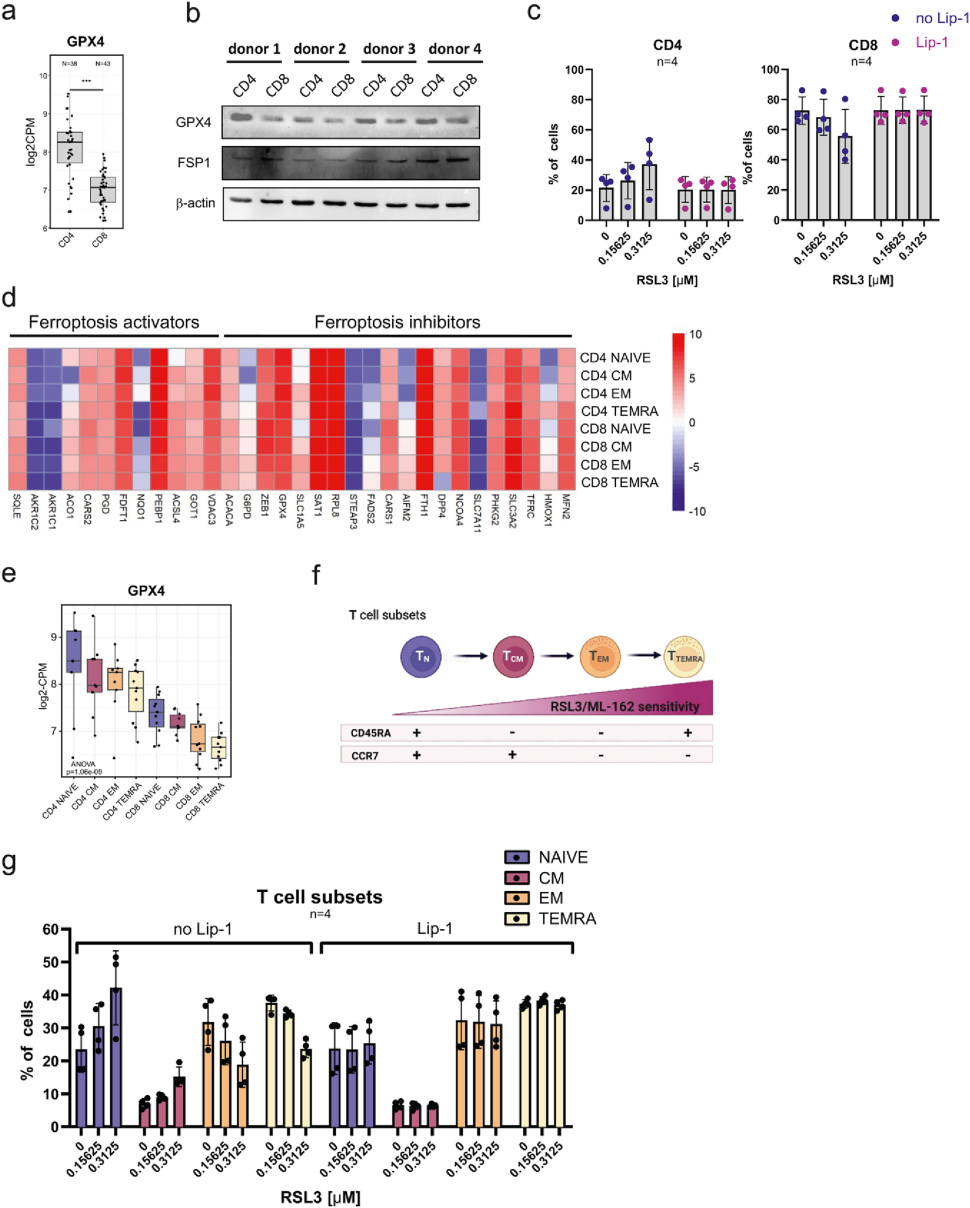
Distinct T cell subsets exhibit differential susceptibility to ferroptosis. **a.** Box plots representing data from RNAseq analysis of GPX4 transcript in CD4^+^ and CD8^+^ T cells (left panel) (dataset: GSE240851); ***p ≤ 0.001. **b.** Western blotting evaluation of GPX4 protein levels in unstimulated CD4 and CD8 T cells. Data show results from four independent donors. β-actin was used as a loading control. **c.** Flow cytometry analysis of the percentages of CD4 (left graph) and CD8 (right graph) T cells after 24 h of RSL3 treatment. Each dot represents an individual donor. Data are presented as means ± sd. **d.** Heatmap of ferroptosis related genes in various T cell subsets. Ferroptosis activating and inhibitory genes were selected based on the study [41]. RNA seq analysis was done using GSE240851 dataset. Row median values of Log2 counts per million (log2 CPM) are included in Suppl. Fig. 3. **e.** Box plots representing data from RNAseq analysis of GPX4 transcript in CD4 + and CD8 + T cells further divided into naïve, central memory (CM), effector memory (EM) and TEMRA subsets (dataset: GSE240851). **f.** Graphical representation of different T cell subsets based on CCR7 and CD45RA expression. Graph adapted from [42]. **g.** Analysis of the percentages of T cell subsets shown in panel (f) after 24 h of RSL3 treatment. The percentage of each subset is calculated relative to the live cell gate. Each dot represents an individual donor. Data are presented as means ± sd. The gating strategy for this analysis is shown in panel Suppl. Fig. 4a

Given the relatively high inter-donor variability in sensitivity to GPX4 inhibition (Fig. 2c), we performed a more detailed analysis to investigate ferroptosis susceptibility across different T cell subsets. To this end, we reanalyzed a publicly available RNA-seq dataset (GSE240851) comprising CD4⁺ and CD8⁺ T cells. We found that GPX4 expression was significantly higher in CD4⁺ than CD8⁺ T cells (Fig. 3a). This phenomenon was further confirmed at the protein level by Western blotting experiments in cell lysates from isolated CD4⁺ and CD8⁺ T cells from 4 healthy donors (Fig. 3b). As a result of transcriptomic and proteomic changes in GPX4 expression in CD4 and CD8 T cells, we performed a standard RSL3 sensitivity assay followed by flow cytometry evaluation of T cell subpopulations after RSL3 treatment (gating strategy Suppl. Fig. 4a). We observed that RSL3 treatment resulted in an increased percentage of CD4^+^ T cells, and a decreased percentage of CD8^+^ T cells, indicating that CD8^+^ T cells are more susceptible to ferroptosis than CD4^+^ T cells. In both cases, Lip-1 treatment completely alleviated the changes observed in CD4⁺ and CD8⁺ subsets (Fig. 3c).

Furthermore, we reanalyzed a publicly available RNA-seq dataset (GSE240851) from peripheral blood mononuclear cells of healthy donors sorted by flow cytometry based on CCR7 and CD45RA expression into naïve, central memory (CM), effector memory (EM), and terminally differentiated effector memory (TEMRA) subsets [26]. In this analysis, ferroptosis activating and inhibitory genes were selected based on the study elucidating ferroptosis in multiple myeloma cells [41]. We observed changes in ferroptosis activating and inhibitory genes between analyzed T cell subsets (Fig. 3d and Suppl. Fig. 3). Among several ferroptosis inhibitors, we focused on GPX4 as one of the most prominent ferroptosis suppressors and demonstrated that decreased GPX4 expression correlated with maturation stages, with the highest levels in naïve subsets, lower levels in CM and EM subsets, and the lowest in TEMRA cells (Fig. 3e). Furthermore, based on the CCR7 and CD45RA staining, we identified four T cell subsets (Fig. 3f) and analyzed their response to RSL3 treatment (Fig. 3g). Our data clearly showed that naïve and CM subsets were less susceptible to RSL3-induced ferroptosis, while EM and TEMRA cells were more sensitive to ferroptosis, as demonstrated by changes in the percentage of these subpopulations upon RSL3 treatment (Fig. 3g). A detailed analysis of each naïve, CM, EM, TEMRA subsets within both CD4^+^ and CD8^+^ T cells revealed a similar pattern of susceptibility to RSL3 treatment (Suppl. Fig. 4b and c). At this step, we concluded that CD4^+^ and CD8^+^ T cells and their different subsets display different sensitivity to ferroptosis that corresponds to their GPX4 expression.

To further better understand the changes in T cells’ ferroptosis sensitivity over long-term culture, we performed a comprehensive analysis of T cells isolated from two healthy donors in terms of their sensitivity to ferroptosis and expression of various surface markers. To this end, cells were stimulated with anti-CD3/CD28 beads and IL-2, and cultured for up to five weeks. Over time, we observed shifts in the proportions of CD4⁺ and CD8⁺ T cells (Suppl. Fig. 4d). At selected time points, we assessed their sensitivity to GPX4 inhibition using RSL3 and ML162 (Suppl. Fig. 4e), and evaluated their phenotypes with the BD Lyoplate™ Human Cell Surface Marker Screening Panel (Suppl. Fig. 4g). Over the course of the culture, a decrease in CD4⁺ T cells, and a corresponding increase in CD8⁺ T cells were observed (Suppl. Fig. 4d). Notably, sensitivity to GPX4 inhibition increased after day 12 post-stimulation and continued to rise over time (Suppl. Fig. 4e). Based on the phenotypic characterization of T cell subsets (Suppl. Fig. 4f), we analyzed the expression of corresponding surface antigens using the screening panel (Suppl. Fig. 4g). The observed trends in surface marker expression support the hypothesis that increased over time ferroptosis susceptibility may be linked to changes in T cell subset composition during in vitro culture.

### GPX4 inhibition decreases CAR-T cells viability and impairs their functions in vitro and in vivo

**Fig. 4:**
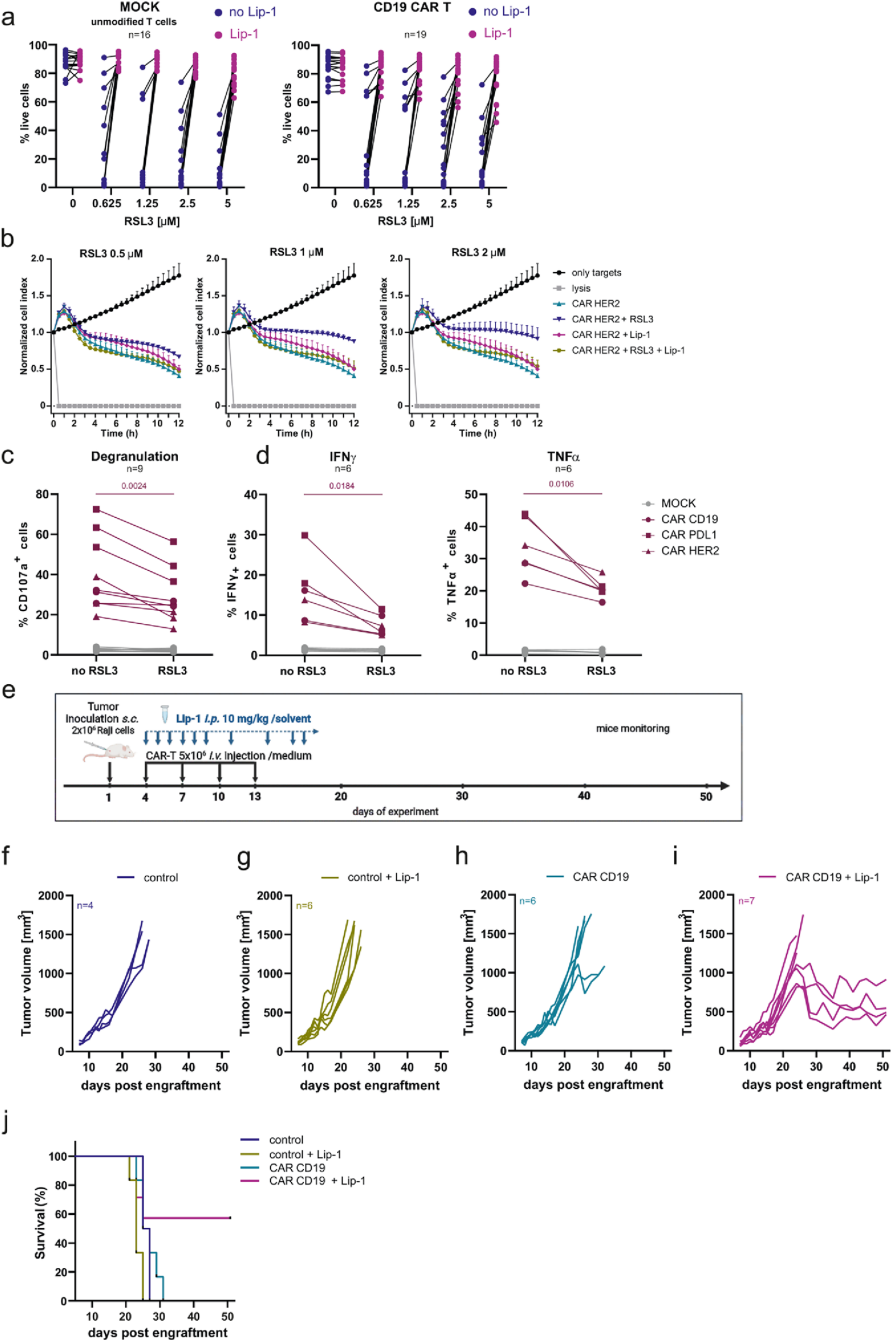
GPX4 inhibition leads to impaired function and reduced antitumor efficacy, which can be restored by Lip-1. **a.** Sensitivity of MOCK (unmodified T cells) and CD19 CAR-T cells to GPX4 inhibition. MOCK or CD19 CAR-T cells were incubated with RSL3 for 48 h in the presence or absence of Lip-1 (0.5 µM). The viability of cells was evaluated using propidium iodide staining followed by flow cytometry analysis. Each data point represents an average of 2 technical replicates for one donor. **b.** Real-time cell analysis of CAR-HER2-mediated killing of MCF7 targets. CAR-T cells were pretreated with RSL3 for 5 h then the cells were added to MCF7 targets and the killing was monitored for the next 12 h. The experiment was done at least 3 times. Representative data from one experiment are presented on the graph. Data represent mean ± sd of 2 technical replicates. **c-d.** Degranulation (**c**) and cytokine production (**d**) IFNy and TNFα of mock unmodified T cells and CAR-T cells in the presence of cancer cells expressing recognized antigen (CD19 CAR-T cells were cocultured with CD19 + Raji cells, CAR HER2 T cells with HER2 + MCF7 and PDL1 CAR-T cells with Raji cells overexpressing PDL1. Effector cells (T cells /CAR-T cells) were pretreated with RSL3 (2.5 µM) for 18 h and subsequently, the cells were tested in a 4 h functional assay. Each data point represents an average of 2 technical replicates for one donor. The statistic was calculated using a paired t-test. **e.** Scheme of the treatment procedures used for in vivo experiments. **f–i.** In vivo results of Lip-1 effect on tumor growth of Raji cells treated with CD19 CAR-T cells. Tumor growth of Raji cells in **f.** control mice, **g.** control mice administrated i.p. with Lip-1 (10 mg/kg), **h.** mice treated with CD19 CAR-T cells, **i.** mice treated with CD19 CAR-T cells in combination with Lip-1 (10 mg/kg) i.p. injections. **j.** Survival rate of mice in four groups: mice inoculated with Raji cells (control), mice inoculated with Raji cells and injected with Lip-1 (control + Lip-1), mice inoculated with Raji cells treated with CD19 CAR-T cells from donor 4 (CAR CD19) and mice inoculated with Raji cells treated with CD19 CAR-T cells and injected with Lip- 1 (CAR CD19 + Lip-1). Each line represents particular mouse

T cells have been widely employed in antitumor immunotherapies after genetic modifications enabling expression of a chimeric antigen receptor (CAR). The manufacturing of CAR-T cells requires the stimulation of T cells before the introduction of the vector encoding CAR receptor. Given our findings that stimulated T cells are prone to ferroptosis, we aimed to assess whether expanded CAR-T cells exhibit the same susceptibility. Accordingly, we observed that CD19 CAR-modified T cells were sensitive to already low concentrations of RSL3 (Fig. 4a and Suppl. Fig. 5a) and ML162 (Suppl. Fig. 5b), similarly to mock (unmodified) T cells, with donor-dependent individual differences. Decreased CAR-T cells’ viability was consistently and almost completely reversible by Lip-1 and ferrostatin 1 (Fer-1).

To understand the functional consequences of increased sensitivity of CAR-T cells to ferroptosis, we assessed the influence of GPX4 inhibitors on CAR-T cells’ cytotoxic activity in both hematological and solid tumor models. In these experiments, CAR-T cells targeting lymphoma (CD19 CAR-T cells), lymphoma overexpressing PD-L1 (PD-L1 CAR-T cells) and breast cancer (HER-2 CAR-T cells) were cultured with cancer cells expressing the corresponding antigens in the presence of RSL3. For the experiments, we selected RSL3 concentrations that were non-toxic to CAR-T cells and only CAR-T cells with at least 80% viability were included in the RTCA analysis (Suppl. Fig. 5c, left panel). Subsequently, we observed that the cytotoxic potential of RSL3-pretreated HER2 CAR-T cells against the MCF7 cell line was impaired compared to the untreated control (Fig. 4b) and unmodified T cells (Suppl. Fig. 5d). This impairment was further reversed by 24-h pretreatment of CAR-T cells with Lip-1 (Fig. 4b) that preserved CAR-T cell viability (Suppl. Fig. 5c, right panel). The viability of MCF7 cells alone in the presence of RSL3 or combination with Lip-1 was unaffected (Suppl. Fig. 5e) and HER2 antigen expression on MCF7 cells remained unchanged following the RSL3 treatment (Suppl. Fig. 5f).

We further demonstrated that pretreatment of CAR-T cells (targeting CD19, PD-L1 or HER-2) with RSL3 at concentrations not toxic to cancer cell lines (Suppl. Fig. 5g) reduced CAR-T cells’ ability to degranulate in response to cognate target cells (Fig. 4c) and to produce IFNγ and TNFα (Fig. 4d).

Finally, we determined the role of ferroptosis in vivo and its influence on the efficacy of CAR-T immunotherapy. To this end, CD19 CAR-T cells were intravenously injected in suboptimal dose into the mouse tail vein in the human-to-mouse Raji xenograft model (Fig. 4e). Before in vivo experiments, the percentage of T cell modification with CAR construct (Suppl. Fig. 5h) and cytotoxic activity of CD19 CAR-T cells against Raji cells were assessed in vitro (Suppl. Fig. 5i). While CD19 CAR-T cells efficiently killed tumor cells in vitro, they were unable to eradicate tumors in vivo (Fig. 4h), as compared with controls (Fig. 4f). Interestingly, antitumor activity of CD19 CAR-T cells was potentiated by Lip-1, as assessed by tumor volume (Fig. 4i) and mouse survival (Fig. 4j). Importantly, Lip-1 alone did not affect tumor cell growth compared to untreated control mice (Fig. 4g).

Taken together, our findings provide compelling evidence that ferroptosis, induced either by pharmacological inactivation of GPX4 in vitro or occurring naturally within the TME in vivo, significantly influences the functionality and cytotoxic activity of CAR-T cells. Furthermore, we also demonstrated that inhibiting ferroptosis using its specific inhibitor, Lip-1, can effectively protect CAR-T cells and enable the effective eradication of cancer cells.

## Discussion

Ferroptosis has recently emerged as an iron-dependent form of regulated cell death that can be induced to overcome the resistance of the tumor cells to existing conventional therapies. An important role in the susceptibility of cancer cells to ferroptosis is played by TME, a multifaceted ecosystem composed of tumor cells, stroma cells and various immune cells. It has been shown that various cellular components of TME (neutrophils, T cells), as well as secreted cytokines (IFNγ, TGFβ1) and metabolic changes (acidosis), have a ferroptosis-promoting role. While the sensitivity of tumor cells to ferroptosis is widely studied, limited data is available how ferroptosis affects the efficacy of immunotherapies dependent on the activation of T cells. Therefore, in this study, we have addressed this issue by comprehensively characterizing ferroptosis markers in T cells. We demonstrated that only after stimulation T cells show hallmarks of ferroptosis [43]. These included an elevated intracellular labile iron pool, increased expression of the iron importer CD71, and an increase of ROS levels [44, 45]. In consequence, in stimulated T cells we observed an increased accumulation of peroxidized lipids, that was mitigated specifically by Lip-1 or Fer-1. While a potential cross-talk between regulated cell death pathways exist, our data do not support the involvement of other forms of cell death, such as apoptosis, necroptosis, pyroptosis, or autophagy, in mediating sensitivity of stimulated T/CAR-T cells to GXP4 inhibition. We acknowledge, however, that validation of additional cell death pathways with complementary assays would further strengthen these conclusions.

Several studies have demonstrated that T cells are relatively resistant to ferroptosis, other research has found T cells to be vulnerable to this form of cell death. Our results are in accordance with report, in which CD8^+^ tumor-infiltrating lymphocytes (TILs) displayed significant amounts of lipid peroxidation.[46] It has been also demonstrated that CD8^+^ T cells can undergo ferroptosis, particularly through mechanisms involving CD36-associated fatty acid intake leading to lipid peroxidation [16, 17]. Drijvers et al. also reported that the inhibition of GPX4 impaired the OT-1 T cells’ antigen-specific killing of cancer cells, and this effect was rescued by ferroptosis inhibitors, vitamin E and Fer-1.[46] Moreover, GPX4 has already been shown to control lipid oxidation and to support physiological T cell responses [47]. In particular, T cells lacking GPX4 failed to expand and to protect mice from acute viral and parasite infections [47]. It was also demonstrated that sensitivity to ferroptosis differs across T cells’ subsets. For example, CD8⁺ T cells are particularly prone to ferroptosis and need GPX4 even for homeostatic survival in peripheral tissues. In T cell–specific Gpx4-deficient mice, it was revealed that CD8⁺ T cells from TΔGpx4/ΔGpx4 mice had an intrinsic defect in maintaining homeostatic balance in the periphery. Moreover, both antigen-specific CD8⁺ and CD4⁺ T cells lacking Gpx4 failed to expand and to protect from viral and parasite infections.[47] CD4⁺ T cells, especially follicular helper T cells, are also vulnerable to ferroptosis post-activation and require GPX4 for survival due to elevated lipid peroxidation [48]. In contrast, Tregs show limited lipid peroxidation compared to tumor-specific CD8^+^ T cells and induction of GPX4 expression upon TCR activation protects them from ferroptosis [49]. However, GPX4 deletion in Tregs can activate ferroptosis, promote IL-1β production, and enhance Th17 cell responses, thereby enhancing antitumor immunity and inhibiting tumor growth [49]. Moreover, Wang et al. demonstrated that RSL3 and erastin treatment did not affect the survival of naïve and shortly stimulated T cells, whereas immunotherapy-activated CD8^+^ T cells induced ferroptosis in tumor cells [18]. From our study, we conclude that CD4⁺ and CD8⁺ cells and their various subsets display different sensitivity to ferroptosis that corresponds to their GPX4 expression. To the best of our knowledge, our observation that CD8⁺ EM and TEMRA cells are the most sensitive to ferroptosis among other CD8⁺ and CD4⁺ subsets was not reported before.

We also provide novel evidence that the sensitivity of T cells to ferroptosis depends on their previous stimulation. Upon TCR stimulation, we observed a significant decrease in the expression level of GPX4 protein that plays a crucial role in protecting cells from lipid peroxidation-induced damage, a phenomenon not described before. We demonstrated that GPX4 decrease can also have important therapeutic consequences. In particular, we determined that GPX4 is essential for the activity of CAR-T cells, as shown by impaired degranulation, cytokine production and killing potential upon GPX4 inhibition. It is widely accepted that the repeated antigen stimulation of CAR-T cells can trigger activation-induced cell death (AICD), which can subsequently reduce the persistence of CAR-T cells and may impact their ability to eliminate tumor cells [50]. However, T cells can also die by oncosis/primary necrosis, a Fas- and caspase-independent, non-apoptotic cell death that occurs in T cell blasts following TCR ligation [51, 52]. Here, we hypothesize that activation-induced ferroptosis could also be the mechanism of CAR-T cells’ fate regulation and maintaining CAR-T cell homeostasis. Accordingly, the induction of ferroptosis in stimulated T/CAR-T cells may occur as part of the contraction phase of an immune response, resulting in the elimination of the effector T cell population while sparing those transformed into long-lived memory cells [53, 54]. This conclusion can be supported by our observations that naïve and CM subsets are less susceptible ferroptosis, while EM and TEMRA cells are more sensitive to this type of death. However, we acknowledge that a more detailed characterization of activated T/CAR-T cells is warranted to further explore the phenomenon of activation-induced ferroptosis.

Finally, our in vivo study demonstrates that inhibiting ferroptosis could be a targeted approach to enhance the efficacy of CAR-T. Our results also indirectly provide support for the presence of ferroptosis within the TME, as was previously described by Kim et al.[12]. Indeed, we observed that the diminished cytotoxic potential of CAR-T cells may only become apparent in vivo, despite decent CAR-T cells efficacy in vitro. Our results indicate that even repeated administration of well-proliferating CAR-T cells is not sufficient when their cytotoxic activity is impaired within TME.

Our findings might also have significant implications for optimizing CAR-T cell therapy and manufacturing. We observed that CAR-T cells, as a result of their stimulation during production, became susceptible to ferroptosis after CAR modification. We hypothesize that increased over time ferroptosis susceptibility may be linked to changes in T cell subset composition during in vitro culture. In the context of CAR-T therapies, this conclusion can shed a new light on the consequences of CAR-T product composition. It is well established that persistence and therapeutic efficacy of CAR-T cells are closely associated with T cells’ differentiation stage [55]. Less-differentiated T cells, such as naïve, and CM subsets, exhibit greater persistence and antitumor efficacy compared to more differentiated populations like EM and TEMRA cells [56]. Both CD8⁺ and CD4⁺ CAR-T cells derived from naïve and CM subsets are more effective relative to those derived from EM subsets [57]. For instance, transcriptomic analyses have shown that CAR-T cells from patients who achieved complete responses in CLL were enriched in a memory-associated gene signature, whereas CAR-T cells from non-responders exhibited gene expression patterns linked to effector differentiation. With our study, we provide additional information on ferroptosis susceptibility as a factor contributing to the persistence and effectiveness of CAR-T cells. We postulate that promoting the less-differentiated phenotype of CAR-T cells should be exploited therapeutically to create CAR-T products characterized by decreased sensitivity to ferroptosis within tumor microenvironment.

Moreover, since susceptibility of T cells/CAR-T cells to ferroptosis increases over time of culture, our study suggests that shortened ex vivo culture period could promote CAR-T resistance to ferroptosis and improve CAR T cell functionality after infusion. Recent extensive studies demonstrated that a shortened ex vivo culture period correlated with improved CAR T cell efficacy[20] and therefore, much effort is being made to reduce the manufacturing time and optimize short-term protocols. Our observations are also consistent with a recent study, in which a substantial accumulation of ROS along with a significant increase in lipid peroxidation was detected in CAR-T cells compared to their pre-culture levels, indicating that manufacturing process induces ferroptosis of CAR-T cells. Accordingly, ferroptosis inhibition during manufacturing significantly enhanced proliferative capacity and cytotoxicity of CAR-T cells [58]. Moreover, it has been recently reported that exposition of CAR-T cells to IL-21 during their manufacturing promotes anti-ferroptosis processes and plays significant roles in enhancing the persistence of CAR-T cells [21].

Altogether, our results indicate that induction of ferroptosis can be a double-edged sword. While the induction of ferroptosis is developed as one of the therapeutic approaches in cancer treatment, particularly for solid tumors, apart from eliminating cancer cells it can also significantly impairs CAR-T cell-mediated antitumor activity. Moreover, post-infusion, the cytotoxic potential of CAR-T cells against tumors is largely constrained by the immunosuppressive TME characterized by oxidative stress, lipid accumulation, and competition for scarce energy resources, rendering immune cells susceptible to ferroptosis [59, 60]. In light of these observations, future anticancer therapies should be carefully designed to selectively induce ferroptosis of tumor cells without impeding CAR-T cells’ antitumor efficacy. Moreover, our study supports the important role of ferroptosis induced during manufacturing process of CAR-T cells in determining their fate and antitumor efficacy.

## Supplementary Information

Below is the link to the electronic supplementary material.

Supplementary file1 (DOCX 15374 kb)

## Data Availability

The data that support the findings of this study are available from the corresponding author, upon reasonable request.

## References

[1] Koeberle SC, Kipp AP, Stuppner H, Koeberle A (2023) Ferroptosis-modulating small molecules for targeting drug-resistant cancer: challenges and opportunities in manipulating redox signaling. Med Res Rev 43:614–682 36658724 10.1002/med.21933 PMC10947485

[2] Zhang C, Liu X, Jin S, Chen Y, Guo R (2022) Ferroptosis in cancer therapy: a novel approach to reversing drug resistance. Mol Cancer 21:47 35151318 10.1186/s12943-022-01530-y PMC8840702

[3] Yin L et al (2022) Ferroptosis-related small-molecule compounds in cancer therapy: strategies and applications. Eur J Med Chem 244:114861 36332549 10.1016/j.ejmech.2022.114861

[4] Li Z et al (2020) Targeting ferroptosis in breast cancer. Biomark Res 8:58 33292585 10.1186/s40364-020-00230-3 PMC7643412

[5] Yang F, Xiao Y, Ding JH, Jin X, Ma D, Li DQ, Shi JX, Huang W, Wang YP, Jiang YZ, Shao ZM (2023) Ferroptosis heterogeneity in triple-negative breast cancer reveals an innovative immunotherapy combination strategy. Cell Metab 35(1):84–100 36257316 10.1016/j.cmet.2022.09.021

[6] Cai Y et al (2023) Alpha-KG inhibits tumor growth of diffuse large B-cell lymphoma by inducing ROS and TP53-mediated ferroptosis. Cell Death Discov 9:182 37308557 10.1038/s41420-023-01475-1 PMC10260963

[7] Li Y et al (2023) Targeting fatty acid synthase modulates sensitivity of hepatocellular carcinoma to sorafenib via ferroptosis. J Exp Clin Cancer Res 42:6 36604718 10.1186/s13046-022-02567-z PMC9817350

[8] Weigand I et al (2020) Active steroid hormone synthesis renders adrenocortical cells highly susceptible to type II ferroptosis induction. Cell Death Dis 11:192 32184394 10.1038/s41419-020-2385-4 PMC7078189

[9] Dixon SJ et al (2012) Ferroptosis: an iron-dependent form of nonapoptotic cell death. Cell 149:1060–1072 22632970 10.1016/j.cell.2012.03.042 PMC3367386

[10] Dixon SJ, Stockwell BR (2014) The role of iron and reactive oxygen species in cell death. Nat Chem Biol 10:9–17 24346035 10.1038/nchembio.1416

[11] Doll S et al (2019) FSP1 is a glutathione-independent ferroptosis suppressor. Nature 575:693–698 31634899 10.1038/s41586-019-1707-0

[12] Kim R et al (2022) Ferroptosis of tumour neutrophils causes immune suppression in cancer. Nature 612:338–346 36385526 10.1038/s41586-022-05443-0 PMC9875862

[13] Zhao Y, Liu Z, Liu G, Zhang Y, Liu S, Gan D, Chang W, Peng X, Sung ES, Gilbert K, Zhu Y (2023) Neutrophils resist ferroptosis and promote breast cancer metastasis through aconitate decarboxylase 1. Cell Metab 35(10):1688–1703 37793345 10.1016/j.cmet.2023.09.004 PMC10558089

[14] Morgan PK et al (2024) A lipid atlas of human and mouse immune cells provides insights into ferroptosis susceptibility. Nat Cell Biol 26:645–659 38589531 10.1038/s41556-024-01377-z

[15] Xu S, Chaudhary O, Rodríguez-Morales P, Sun X, Chen D, Zappasodi R, Xu Z, Pinto AF, Williams A, Schulze I, Farsakoglu Y (2021) Uptake of oxidized lipids by the scavenger receptor CD36 promotes lipid peroxidation and dysfunction in CD8+ T cells in tumors. Immunity 54(7):1561–1577 34102100 10.1016/j.immuni.2021.05.003 PMC9273026

[16] Ma X, Xiao L, Liu L, Ye L, Su P, Bi E, Wang Q, Yang M, Qian J, Yi Q (2021) CD36-mediated ferroptosis dampens intratumoral CD8+ T cell effector function and impairs their antitumor ability. Cell Metab 33(5):1001–1012 33691090 10.1016/j.cmet.2021.02.015 PMC8102368

[17] Han C et al (2024) Cystine deprivation triggers CD36-mediated ferroptosis and dysfunction of tumor infiltrating CD8(+) T cells. Cell Death Dis 15:145 38360744 10.1038/s41419-024-06503-1 PMC10869360

[18] Wang W et al (2019) CD8(+) T cells regulate tumour ferroptosis during cancer immunotherapy. Nature 569:270–274 31043744 10.1038/s41586-019-1170-y PMC6533917

[19] Chen DS, Mellman I (2013) Oncology meets immunology: the cancer-immunity cycle. Immunity 39:1–10 23890059 10.1016/j.immuni.2013.07.012

[20] Ghassemi S et al (2018) Reducing ex vivo culture improves the antileukemic activity of chimeric antigen receptor (CAR) T cells. Cancer Immunol Res 6:1100–1109 30030295 10.1158/2326-6066.CIR-17-0405 PMC8274631

[21] Zhang M et al (2024) Optimizing CAR-T cell culture: differential effects of IL-2, IL-12, and IL-21 on CAR-T cells. Cytokine 184:156758 39299100 10.1016/j.cyto.2024.156758

[22] Bajor M et al (2018) Targeting peroxiredoxin 1 impairs growth of breast cancer cells and potently sensitises these cells to prooxidant agents. Br J Cancer 119:873–884 30287919 10.1038/s41416-018-0263-y PMC6189216

[23] Bajor M et al (2022) PD-L1 CAR effector cells induce self-amplifying cytotoxic effects against target cells. J Immunother Cancer. 10.1136/jitc-2021-002500 35078921 10.1136/jitc-2021-002500 PMC8796262

[24] Charan J, Kantharia ND (2013) How to calculate sample size in animal studies? J Pharmacol Pharmacother 4:303–306 24250214 10.4103/0976-500X.119726 PMC3826013

[25] Komori HK, Hart T, LaMere SA, Chew PV, Salomon DR (2015) Defining CD4 T cell memory by the epigenetic landscape of CpG DNA methylation. J Immunol Baltim Md 1950(194):1565–1579 10.4049/jimmunol.1401162 PMC4364524 25576597

[26] Khajavi L et al (2023) The transcriptomics profiling of blood CD4 and CD8 T-cells in narcolepsy type I. Front Immunol 14:1249405 38077397 10.3389/fimmu.2023.1249405 PMC10702585

[27] Wingett SW, Andrews S (2018) Fastq screen: a tool for multi-genome mapping and quality control. F1000Res 7:1338 30254741 10.12688/f1000research.15931.1 PMC6124377

[28] Bolger AM, Lohse M, Usadel B (2014) Trimmomatic: a flexible trimmer for Illumina sequence data. Bioinformatics 30:2114–2120 24695404 10.1093/bioinformatics/btu170 PMC4103590

[29] Liao Y, Smyth GK, Shi W (2019) The R package Rsubread is easier, faster, cheaper and better for alignment and quantification of RNA sequencing reads. Nucleic Acids Res 47:e47 30783653 10.1093/nar/gkz114 PMC6486549

[30] Danecek P et al (2021) Twelve years of SAMtools and BCFtools. Gigascience. 10.1093/gigascience/giab008 33590861 10.1093/gigascience/giab008 PMC7931819

[31] Ritchie ME et al (2015) Limma powers differential expression analyses for RNA-sequencing and microarray studies. Nucleic Acids Res 43:e47 25605792 10.1093/nar/gkv007 PMC4402510

[32] Stockwell BR (2022) Ferroptosis turns 10: emerging mechanisms, physiological functions, and therapeutic applications. Cell 185:2401–2421 35803244 10.1016/j.cell.2022.06.003 PMC9273022

[33] Li FJ et al (2022) System X(c) (-)/GSH/GPX4 axis: an important antioxidant system for the ferroptosis in drug-resistant solid tumor therapy. Front Pharmacol 13:910292 36105219 10.3389/fphar.2022.910292 PMC9465090

[34] Yang WS et al (2016) Peroxidation of polyunsaturated fatty acids by lipoxygenases drives ferroptosis. Proc Natl Acad Sci U A 113:E4966–E4975 10.1073/pnas.1603244113 PMC5003261 27506793

[35] Doll S et al (2017) ACSL4 dictates ferroptosis sensitivity by shaping cellular lipid composition. Nat Chem Biol 13:91–98 27842070 10.1038/nchembio.2239 PMC5610546

[36] Dixon SJ et al (2015) Human haploid cell genetics reveals roles for lipid metabolism genes in nonapoptotic cell death. ACS Chem Biol 10:1604–1609 25965523 10.1021/acschembio.5b00245 PMC4509420

[37] Kagan VE et al (2017) Oxidized arachidonic and adrenic PEs navigate cells to ferroptosis. Nat Chem Biol 13:81–90 27842066 10.1038/nchembio.2238 PMC5506843

[38] da XavierSilva TN, Schulte C, Alves AN, Maric HM, Friedmann Angeli JP (2023) Molecular characterization of AIFM2/FSP1 inhibition by iFSP1-like molecules. Cell Death Dis 14(4):281 37080964 10.1038/s41419-023-05787-z PMC10119282

[39] Lee S et al (2023) Autophagy mediates an amplification loop during ferroptosis. Cell Death Dis 14:464 37491375 10.1038/s41419-023-05978-8 PMC10368698

[40] Merkley SD, Chock CJ, Yang XO, Harris J, Castillo EF (2018) Modulating T cell responses via autophagy: the intrinsic influence controlling the function of both antigen-presenting cells and T cells. Front Immunol 9:2914 30619278 10.3389/fimmu.2018.02914 PMC6302218

[41] Xian M et al (2024) Leukocyte immunoglobulin-like receptor B1 (LILRB1) protects human multiple myeloma cells from ferroptosis by maintaining cholesterol homeostasis. Nat Commun 15:5767 38982045 10.1038/s41467-024-50073-x PMC11233649

[42] Lugli E, Gattinoni L (2015) Harnessing stem cell-like memory T cells for adoptive cell transfer therapy of cancer. InDevelopments in T Cell Based Cancer Immunotherapies. Springer International Publishing. Cham (pp. 183–209). 10.1007/978-3-319-21167-1_8.

[43] Stockwell BR (2019) The hallmarks of ferroptosis. Annu Rev Cancer Biol 3:35 41613499 10.1146/annurev-cancerbio-030518-055844 PMC12851585

[44] Simeoni L, Bogeski I (2015) Redox regulation of T-cell receptor signaling. Biol Chem 396:555–568 25781677 10.1515/hsz-2014-0312

[45] Feng H, Schorpp K, Jin J, Yozwiak CE, Hoffstrom BG, Decker AM, Rajbhandari P, Stokes ME, Bender HG, Csuka JM, Upadhyayula PS (2020) Transferrin receptor is a specific ferroptosis marker. Cell Rep 30(10):3411–3423 32160546 10.1016/j.celrep.2020.02.049 PMC7172030

[46] Drijvers JM et al (2021) Pharmacologic Screening Identifies Metabolic Vulnerabilities of CD8(+) T Cells. Cancer Immunol Res 9:184–199 33277233 10.1158/2326-6066.CIR-20-0384 PMC7864883

[47] Matsushita M et al (2015) T cell lipid peroxidation induces ferroptosis and prevents immunity to infection. J Exp Med 212:555–568 25824823 10.1084/jem.20140857 PMC4387287

[48] Yao Y et al (2021) Selenium-GPX4 axis protects follicular helper T cells from ferroptosis. Nat Immunol 22:1127–1139 34413521 10.1038/s41590-021-00996-0

[49] Xu C et al (2021) The glutathione peroxidase Gpx4 prevents lipid peroxidation and ferroptosis to sustain Treg cell activation and suppression of antitumor immunity. Cell Rep 35:109235 34133924 10.1016/j.celrep.2021.109235

[50] Huan T et al (2022) Activation-induced cell death in CAR-T cell therapy. Hum Cell 35:441–447 35032297 10.1007/s13577-022-00670-z

[51] Inaba M et al (1999) Primed T cells are more resistant to Fas-mediated activation-induced cell death than naive T cells. J Immunol 163:1315–1320 10415029

[52] Davidson WF, Haudenschild C, Kwon J, Williams MS (2002) T cell receptor ligation triggers novel nonapoptotic cell death pathways that are Fas-independent or Fas-dependent. J Immunol 169:6218–6230 12444127 10.4049/jimmunol.169.11.6218

[53] Badovinac VP, Porter BB, Harty JT (2002) Programmed contraction of CD8(+) T cells after infection. Nat Immunol 3:619–626 12055624 10.1038/ni804

[54] Garrod KR et al (2012) Dissecting T cell contraction in vivo using a genetically encoded reporter of apoptosis. Cell Rep 2:1438–1447 23159042 10.1016/j.celrep.2012.10.015

[55] Zhang H, Zhao P, Huang H (2020) Engineering better chimeric antigen receptor T cells. Exp Hematol Oncol 9:34 33292660 10.1186/s40164-020-00190-2 PMC7709221

[56] Knochelmann HM et al (2018) CAR t cells in solid tumors: blueprints for building effective therapies. Front Immunol 9:1740 30140266 10.3389/fimmu.2018.01740 PMC6094980

[57] Turtle CJ et al (2016) CD19 CAR–T cells of defined CD4+:CD8+ composition in adult B cell ALL patients. J Clin Invest 126:2123–2138 27111235 10.1172/JCI85309 PMC4887159

[58] Harada S et al (2023) Ferroptosis inhibition generates TCF-1 + CAR-T cells with enhanced persistence and cytotoxicity. Blood 142:97–97

[59] Curvello R, Berndt N, Hauser S, Loessner D (2024) Recreating metabolic interactions of the tumour microenvironment. Trends Endocrinol Metab. 10.1016/j.tem.2023.12.005 10.1016/j.tem.2023.12.005 38212233

[60] Nanjireddy PM, Olejniczak SH, Buxbaum NP (2023) Targeting of chimeric antigen receptor T cell metabolism to improve therapeutic outcomes. Front Immunol 14:1121565 36999013 10.3389/fimmu.2023.1121565 PMC10043186

