# Correction to: GPX4 is a key ferroptosis regulator orchestrating T cells and CAR-T-cells sensitivity to ferroptosis

**DOI:** 10.1007/s00262-025-04194-x

**Published:** 2026-04-07

**Authors:** Marta Kłopotowska, Iwona Baranowska, Szymon Hajduk, Anna Jurga, Natalia Leśniowska, Michał Łaźniewski, Monika Granica, Marta Krawczyk, Milena Dziewicka, Agnieszka Graczyk, Jan Słupski, Radosław Zagożdżon, Dariusz Plewczynski, Magdalena Winiarska, Malgorzata Bajor

**Affiliations:** 1 Department of Immunology, Mossakowski Medical Research Institute Polish Academy of Sciences, 5 Adolfa Pawinskiego St., 02-106 Warsaw, Poland; 2 Immunooncology Students’ Science Association, Medical University of Warsaw, Warsaw, Poland; 3 Department of Bacteriology and Biocontamination Control, National Institute of Public Health NIH—National Research Institute, 24 Chocimska St., 00-791 Warsaw, Poland; 4 Centre for Advanced Materials and Technologies, Warsaw University of Technology, 19 Poleczki St., 02-822 Warsaw, Poland; 5 Department of Immunology, Medical University of Warsaw, 5 Jana Nielubowicza St., 02-097 Warsaw, Poland; 6 Doctoral School, Medical University of Warsaw, 81 Zwirki I Wigury St., 02-091 Warsaw, Poland; 7 Doctoral School of Translational Medicine, Mossakowski Medical Research Institute, Polish Academy of Sciences, 5 Adolfa Pawinskiego St., 02-106 Warsaw, Poland; 8 Laboratory of Cellular and Genetic Therapies, Medical University of Warsaw, 02-097 Warsaw, Poland; 9 Centre of New Technologies, University of Warsaw, 2C Banacha St., 02-097 Warsaw, Poland; 10 Faculty of Mathematics and Information Science, Warsaw University of Technology, 75 Koszykowa St., 00-662 Warsaw, Poland

**Correction to: Cancer Immunology, Immunotherapy (2025) 74:280** 10.1007/s00262-025-04133-w

In the original version of this article, the affiliations were linked to the authors incorrectly as

Marta Kłopotowska^1^ · Iwona Baranowska1 · Szymon Hajduk^1,2^ · Anna Jurga^1,2^ · Natalia Leśniowska^1,2^ · Michał Łaźniewski^5,6^ · Monika Granica^1,3,4^ · Marta Krawczyk^1,7^ · Milena Dziewicka^1,2^ · Agnieszka Graczyk^1^ · Jan Słupski^1,2^ · Radosław Zagożdżon^8^ · Dariusz Plewczynski^9,10^ · Magdalena Winiarska^1,3^ · Malgorzata Bajor^1^

^1^Department of Immunology, Mossakowski Medical Research Institute Polish Academy of Sciences, 5 Adolfa Pawinskiego St., 02‑106 Warsaw, Poland

^2^Immunooncology Students’ Science Association, Medical University of Warsaw, Warsaw, Poland

^3^Department of Bacteriology and Biocontamination Control, National Institute of Public Health NIH—National Research Institute, 24 Chocimska St., 00‑791 Warsaw, Poland

^4^Centre for Advanced Materials and Technologies, Warsaw University of Technology, 19 Poleczki St., 02‑822 Warsaw, Poland

^5^Department of Immunology, Medical University of Warsaw, 5 Jana Nielubowicza St., 02‑097 Warsaw, Poland

^6^Doctoral School, Medical University of Warsaw, 81 Zwirki I Wigury St., 02‑091 Warsaw, Poland

^7^Doctoral School of Translational Medicine, Mossakowski Medical Research Institute, Polish Academy of Sciences, 5 Adolfa Pawinskiego St., 02‑106 Warsaw, Poland

^8^Laboratory of Cellular and Genetic Therapies, Medical University of Warsaw, 02‑097 Warsaw, Poland

^9^Centre of New Technologies, University of Warsaw, 2C Banacha St., 02‑097 Warsaw, Poland

^10^Faculty of Mathematics and Information Science, Warsaw University of Technology, 75 Koszykowa St., 00‑662 Warsaw, Poland

but should have been

Marta Kłopotowska^1^, Iwona Baranowska^1^, Szymon Hajduk^1,2^, Anna Jurga^1,2^, Natalia Leśniowska^1,2^, Michał Łaźniewski^3,4^, Monika Granica^1,5,6^, Marta Krawczyk^1,7^, Milena Dziewicka^1,2^, Agnieszka Graczyk^1^, Jan Słupski^1,2^, Radosław Zagożdżon^8^, Dariusz Plewczynski^9,10^, Magdalena Winiarska^1,5^, Malgorzata Bajor^1^

^1^Department of Immunology, Mossakowski Medical Research Institute Polish Academy of Sciences, 5 Adolfa Pawinskiego St., 02-106 Warsaw, Poland

^2^Immunooncology Students’ Science Association, Medical University of Warsaw, Warsaw, Poland

^3^Department of Bacteriology and Biocontamination Control, National Institute of Public Health NIH—National Research Institute, 24 Chocimska St., 00-791 Warsaw, Polan

^4^Centre for Advanced Materials and Technologies, Warsaw University of Technology, 19 Poleczki St., 02-822 Warsaw, Poland

^5^Department of Immunology, Medical University of Warsaw, 5 Jana Nielubowicza St., 02-097 Warsaw, Poland

^6^Doctoral School, Medical University of Warsaw, 81 Zwirki i Wigury St., 02-091 Warsaw, Poland

^7^Doctoral School of Translational Medicine, Mossakowski Medical Research Institute, Polish Academy of Sciences, 5 Adolfa Pawinskiego St., 02-106 Warsaw, Poland

^8^Laboratory of Cellular and Genetic Therapies, Medical University of Warsaw, 02-097 Warsaw, Poland

^9^Centre of New Technologies, University of Warsaw, 2C Banacha St., 02-097 Warsaw, Poland

^10^Faculty of Mathematics and Information Science, Warsaw University of Technology, 75 Koszykowa St., 00-662 Warsaw, Poland

