# Supplementary material for: GPX4 is a key ferroptosis regulator orchestrating T cells and CAR-T-cells sensitivity to ferroptosis
Source: Cancer Immunol Immunother. 2025 Aug 4;74(9):280. doi: 10.1007/s00262-025-04133-w (PMC12321709; doi:10.1007/s00262-025-04133-w)
Supplement: Supplementary file 1 — Supplementary file1 (DOCX 15374 kb) [file 262_2025_4133_MOESM1_ESM.docx]

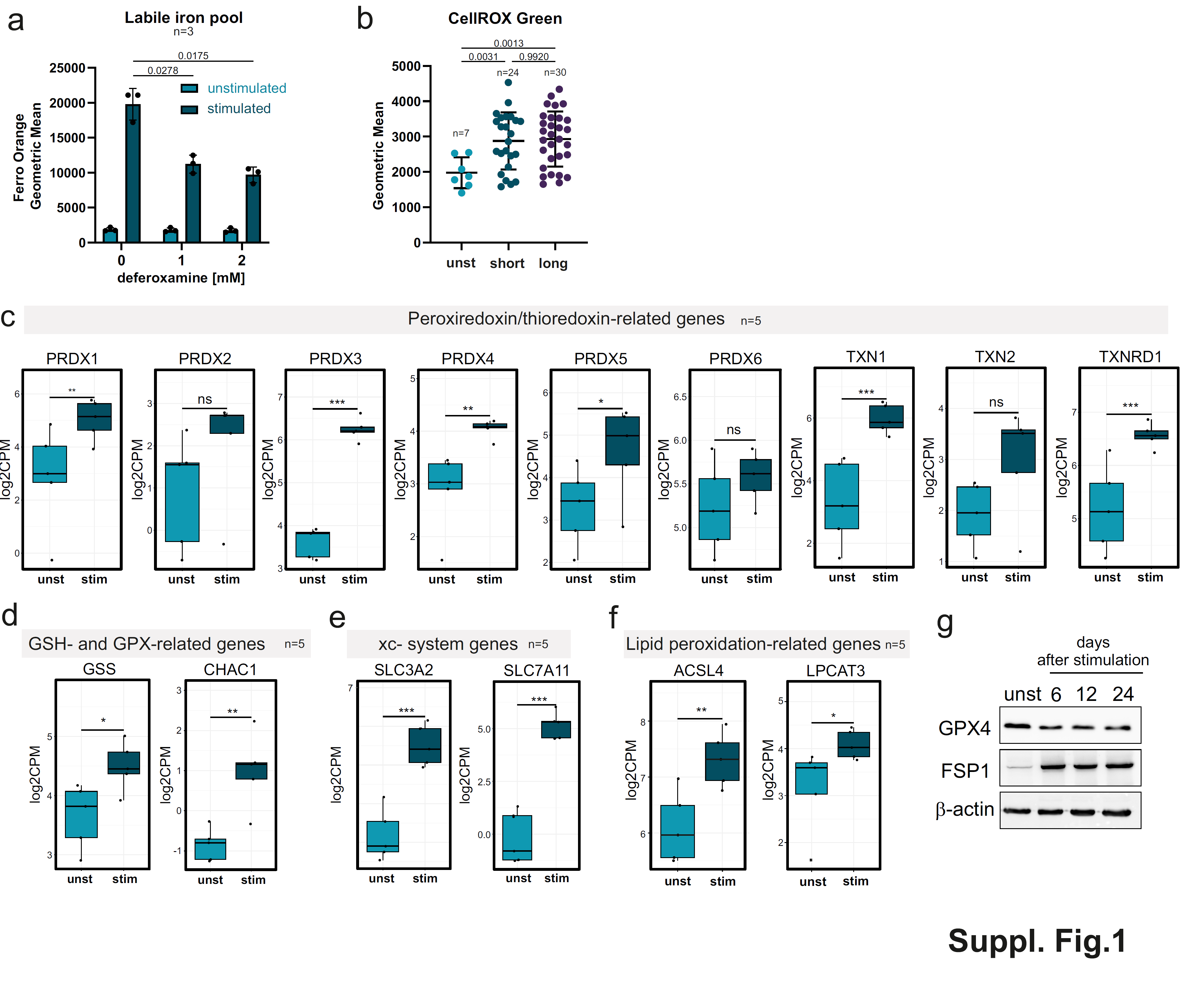


**Suppl. Fig. 1. Stimulation of T cells alters the expression levels of transcripts encoding proteins that regulate ferroptosis.**

**a.** FerroOrange staining of labile iron pool in unstimulated T cells and stimulated with anti-CD3/anti-CD28 Dynabeads and IL-2 for 3 days. The cells were washed 3 times and resuspended in HBSS buffer in the presence or absence of 1 mM or 2 mM deferoxamine for 30 min before FerroOrange staining.

**b.** Intracellular ROS detection using fluorescent probe CellROX Green and flow cytometry analysis. Statistical analysis was done with Brown-Forsythe and Welch ANOVA tests with Dunnett; T3 multiple comparisons test, with individual variances computed for each comparison.

**c.** RNAseq analysis of transcripts for peroxiredoxin/thioredoxin-related genes: PRDX1-6 (peroxiredoxins 1-6), TXN 1, 2 (thioredoxins 1, 2) and thioredoxin reductase TXNRD1 in unstimulated CD4+ T cells and stimulated with CD3/CD28 beads for 48 h (GEO accession number: GSE 59846); * p ≤ 0.05; ** p ≤ 0.01; *** p ≤ 0.001; ns: not significant.

**d.** RNA-seq analysis of transcripts of glutathione synthetase (GSS), glutathione-specific gamma-glutamylcyclotransferase 1 (CHAC1) in unstimulated CD4+ T cells and stimulated with CD3/CD28 beads for 48 h (GEO accession number: GSE 59846); * p ≤ 0.05; ** p ≤ 0.01.

**e.** RNA-seq analysis of xc- related transcripts: amino acid transporter heavy chain (SLC3A2), cystine/glutamate transporter (SLC7A11) in unstimulated CD4+ T cells and stimulated with CD3/CD28 beads for 48 h; *** p ≤ 0.001.

**f.** RNA-seq analysis of lipid peroxidation-related transcripts named Long-chain-fatty-acid-CoA ligase 4 (ACSL4) and lysophosphatidylcholine acyltransferase 3 (LPCAT3), in unstimulated CD4+ T cells and stimulated with CD3/CD28 beads for 48 h; * p ≤ 0.05; ** p ≤ 0.01.

**g.** Western blotting analysis of GPX4 and FSP1 protein levels in unstimulated and stimulated T cells at different days upon stimulation. Data show results from another representative donor. β-actin was used as a loading control.


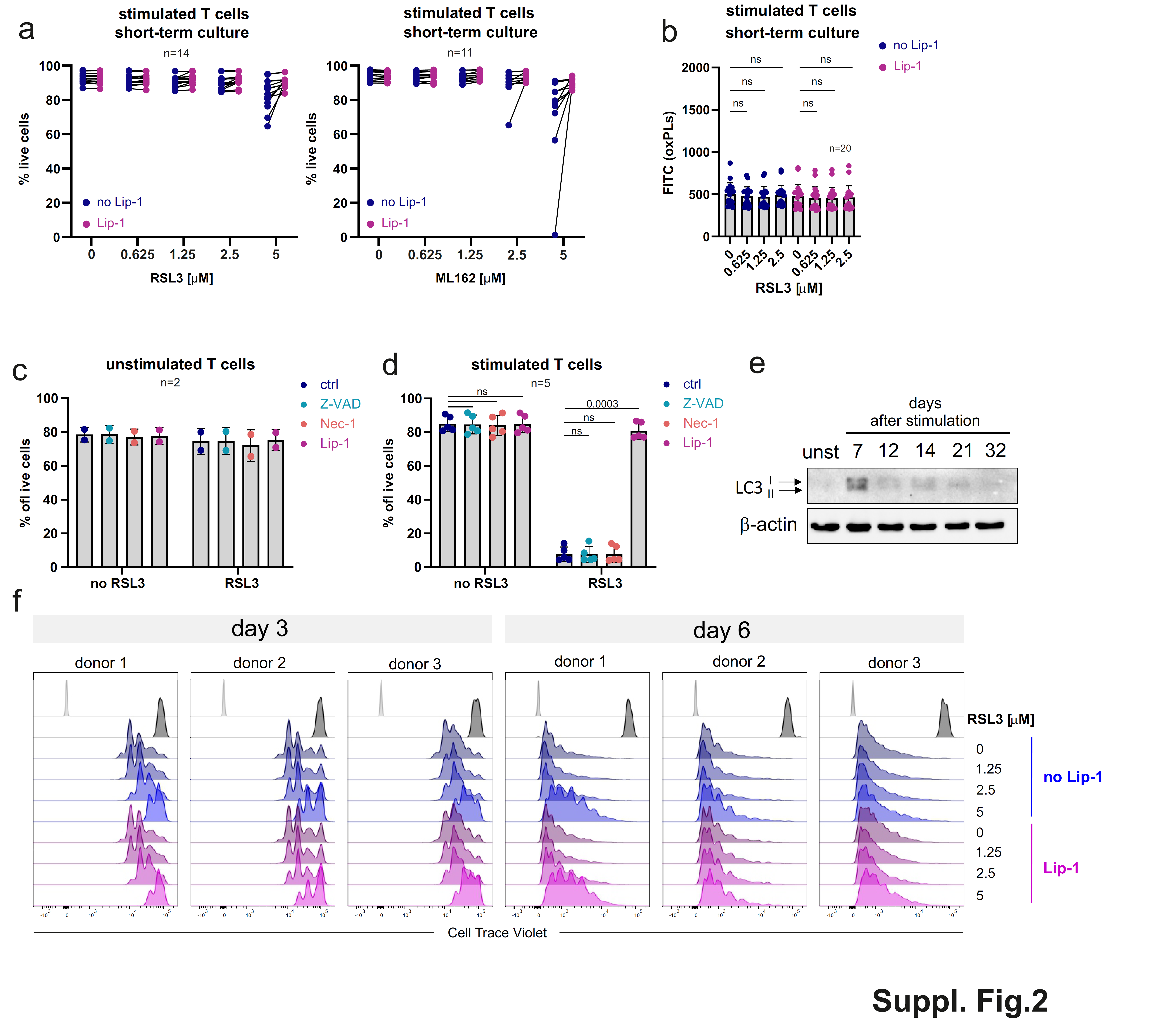


**Suppl. Fig. 2. GPX4 inhibition neither induce other types of cell death in unstimulated and stimulated T cells nor affects T cell proliferation.**

**a.** Sensitivity of short term-culture stimulated T cells to GPX4 inhibition. Human primary T cells were stimulated with (CD3/CD28 beads) and IL-2 (100 U/ml). Subsequently, T cells were cultured shortly, less than 2 weeks and were seeded with RSL3 or ML162 for 48 h in the presence or absence of Lip-1 (0.5 µM). Each data point represents an average of 2 technical replicates for one donor.

**b.** Lipid peroxidation of stimulated T cells after short term-culture (less than 2 weeks) upon 24 h RSL3 treatment; evaluated with C11-BODIPY 581/591 staining. Each data point represents averages of 2 technical replicates for one donor. Data are presented as means +/- sd. Statistical analysis was done with ordinary two-way ANOVA with Dunnett's multiple comparisons test; ns: not significant.

**c, d.** Survival of unstimulated (a) and stimulated (b) T cells in the presence or absence of RSL3 (2.5 µM) in combination with an inhibitor of apoptosis (Z-VAD, 10 µM), necroptosis (Nec-1, 10 µM) and ferroptosis (Lip-1, 0.5 µM). The statistic was calculated with one-way ANOVA with repeated measures and Geisser-Greenhouse correction with Sidak’s multiple comparisons test; ns: not significant.

**e.** Western blotting evaluation of LC3 protein level in unstimulated and stimulated T cells at different days upon stimulation. Data show results from 1 representative donor. β-actin was used as a loading control.

**f.** Proliferation of T cells stimulated with CD3/CD28 beads and IL-2 in the presence of increasing concentrations of RSL3 (blue histograms) or RSL3 and Lip-1 (pink histograms). Proliferation was evaluated upon Cell Trace Violet staining and flow cytometry analysis. Data are presented from 3 donors after 3 days of stimulation (left panel) and 6 days of stimulation (right panel). Unstained control was marked as light grey histogram, CTV-positive unstimulated (non-proliferating) control (dark grey).


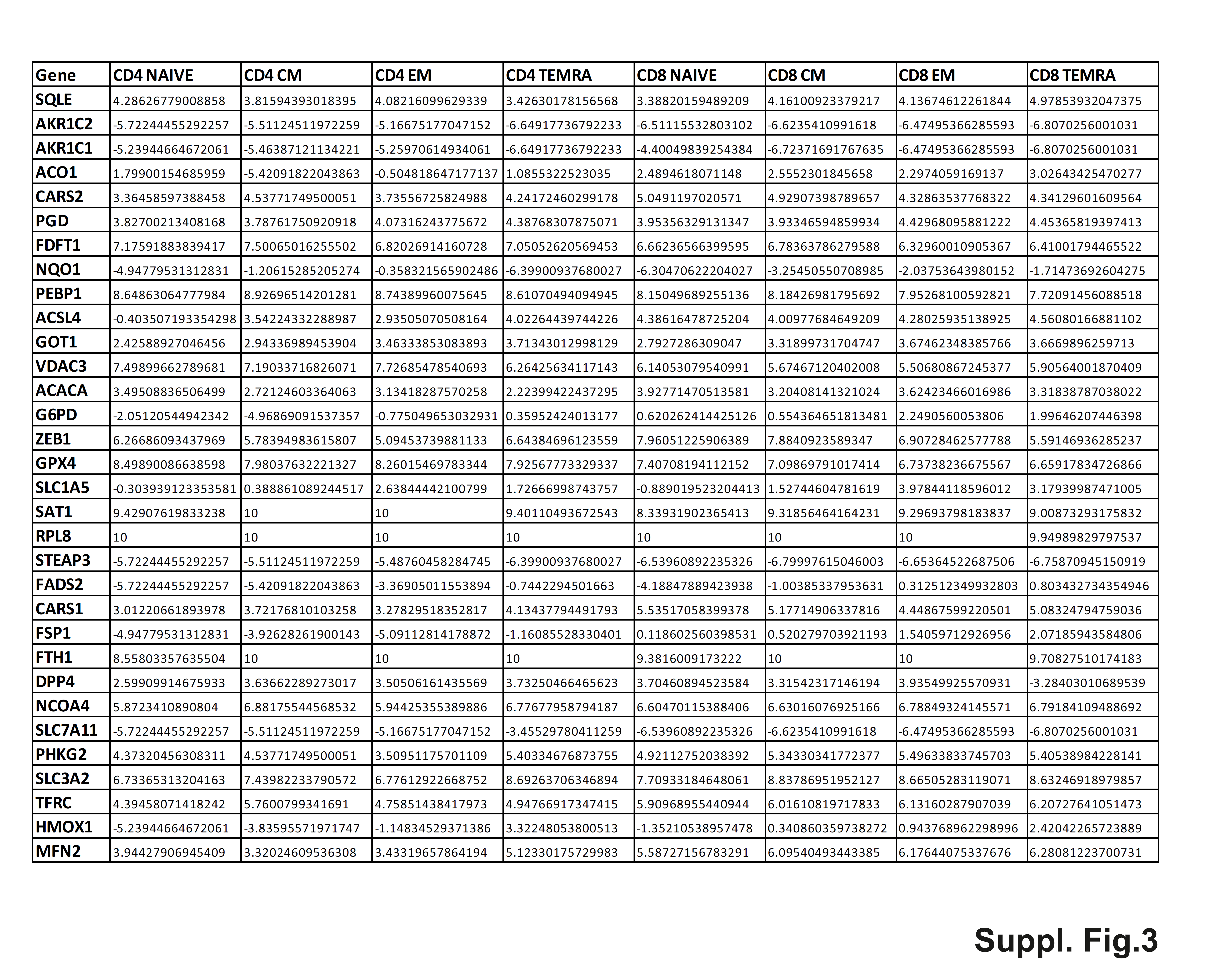


**Suppl. Fig. 3.** Table showing median values of Log2 counts per million (log2 CPM) corresponding to the heatmap presented in Fig. 3d.


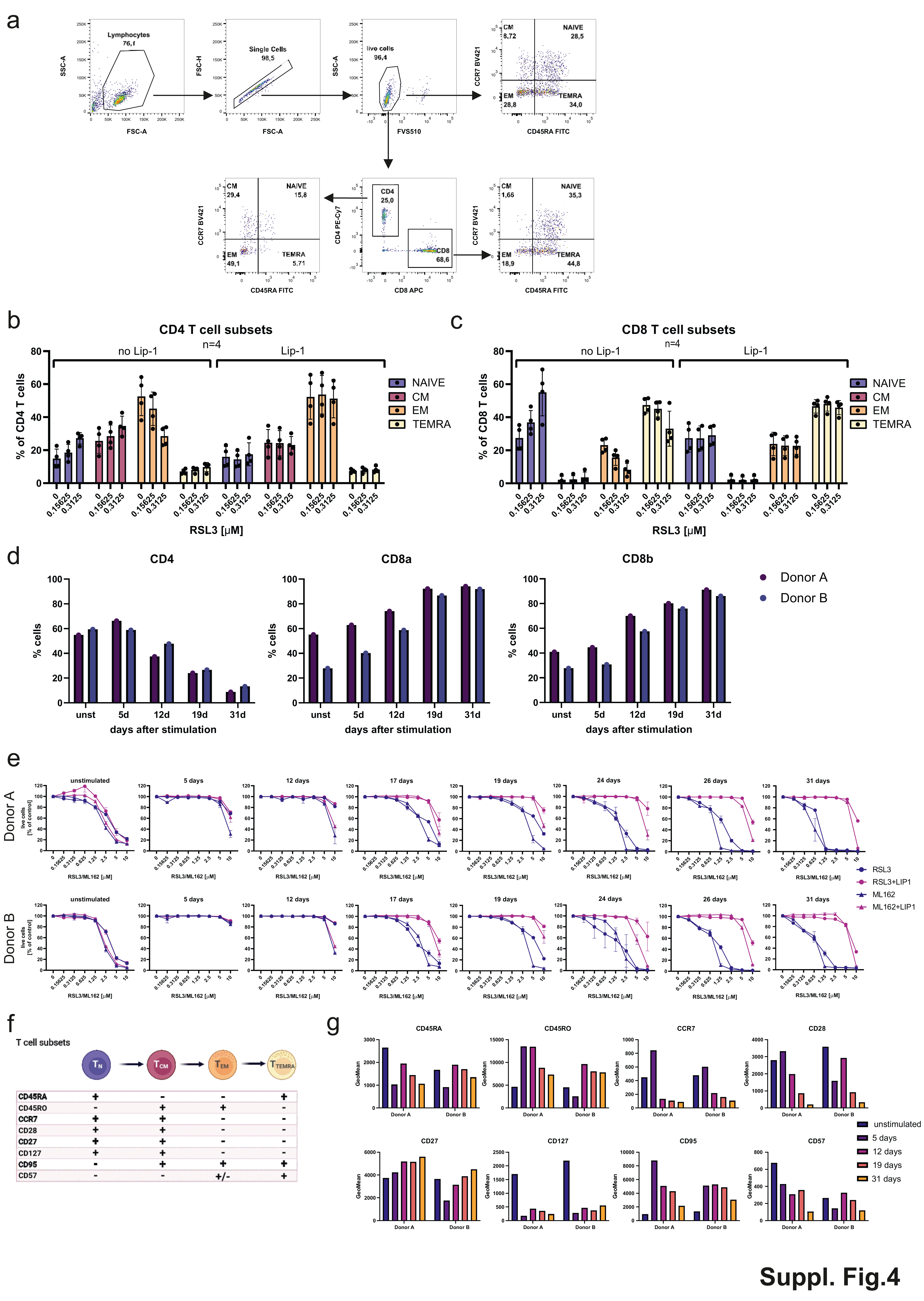


**Suppl. Fig. 4. T cells undergo changes in subset composition over the course of in vitro culture and alter their sensitivity to ferroptosis.**

**a.** Gating strategy used for flow cytometry analysis of T cell subsets.

**b, c.** Analysis of the percentages of T cell subsets based on the expression of CCR7 and CD45RA after 24 h of RSL3 treatment determined within **b.** CD4^+^ or **c.** CD8^+^ populations. Each dot represents an individual donor. Data are presented as means +/- sd. The gating strategy for this analysis is shown in panel Suppl. Fig. 4a.

**d.** Flow cytometry analysis of the percentages of CD4^+^ and CD8^+^ T cells in unstimulated and stimulated T cells at specific days post-stimulation. Staining was performed using the BD Lyoplate™ Human Cell Surface Marker Screening Panel.

**e.** Analysis of sensitivity to GPX4 inhibitors RSL3 and ML162 in unstimulated and stimulated T cells on various days following stimulation. Donor A (upper panel) and donor B (lower panel).

**f.** Scheme representing diversity and functionality of human peripheral T cell subsets, modified version adapted from^42^

**g.** Flow cytometry analysis of selected surface antigens (presented in panel f.) on unstimulated and stimulated T cells at specific time points post-stimulation, performed using the BD Lyoplate™ Human Cell Surface Marker Screening Panel. Data are presented as geometric means.


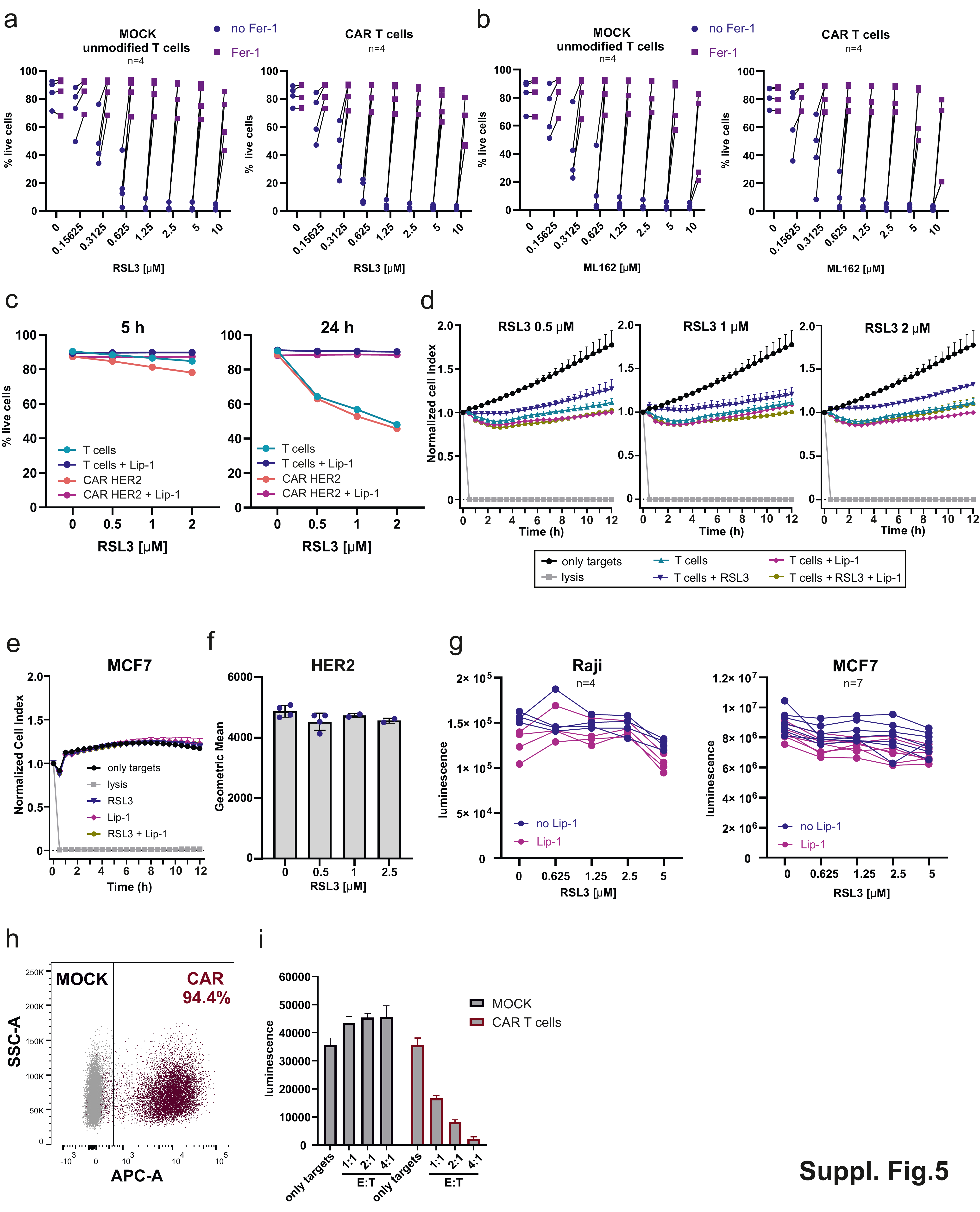


**Suppl. Fig. 5. Evaluation of survival and cytotoxicity in effector cells under GPX4 inhibition.**

**a, b.** Sensitivity of MOCK (unmodified T cells) and CD19 CAR-T cells to GPX4 inhibition reversed by ferrostatin-1 (Fer-1) pretreatment. MOCK or CD19 CAR-T cells were preincubated with 5 µM Fer-1 for 24h and subsequently seeded with increasing concentrations of **a.** RSL3 or **b.** ML162 for the next 48 h. The viability of cells was evaluated using propidium iodide staining followed by flow cytometry analysis. Each data point represents an average of 2 technical replicates for one donor.

**c.** Viability of T cells and CAR-HER2 T cells treated with RSL3 (with or without Lip-1) for 5 h and 24 h, evaluated with propidium iodide staining and flow cytometry analysis.

**d.** Supplementary data for the main figure 4b showing results of real-time cell analysis of control unmodified T cell killing of MCF7 targets. T cells were pretreated with RSL3 for 5 h then the cells were added to MCF7 targets and assay was monitored for the next 12 hours. Experiment was repeated at least 3 times, data represent mean and standard deviation of 2 technical replicates from one representative experiment.

**e.** RTCA results of the impact of RSL3 on MCF7 cells proliferation.

**f.** Expression of HER2 antigen on MCF7 target cells preincubated with RSL3. MCF7 cells were seeded onto a 12-well plate and allowed to adhere overnight. The next day RSL3 was added to appropriate wells, and after 24 h MCF7 cells were trypsinized, stained with viability stain and anti-HER2 antibody and analyzed on a flow cytometer.

**g.** Raji and MCF7 cells, previously modified to express the luciferase reporter gene (red-luc), were seeded with RSL3 for 48 h in the presence or absence of Lip-1 (0.5 µM). After incubation, Bright-Glo™ Luciferase Assay System was used for bioluminescence readout.

**h.** Dot plots from flow cytometry analysis showing CD19 CAR expression (burgundy color) in modified CAR-T cells. MOCK - control unmodified T cells are marked in grey.

**i.** CD19 CAR-T cell-mediated killing of Raji red-luc cells evaluated with luminescence-based assay. Data are presented as averages from 4 technical replicates +/- sd.


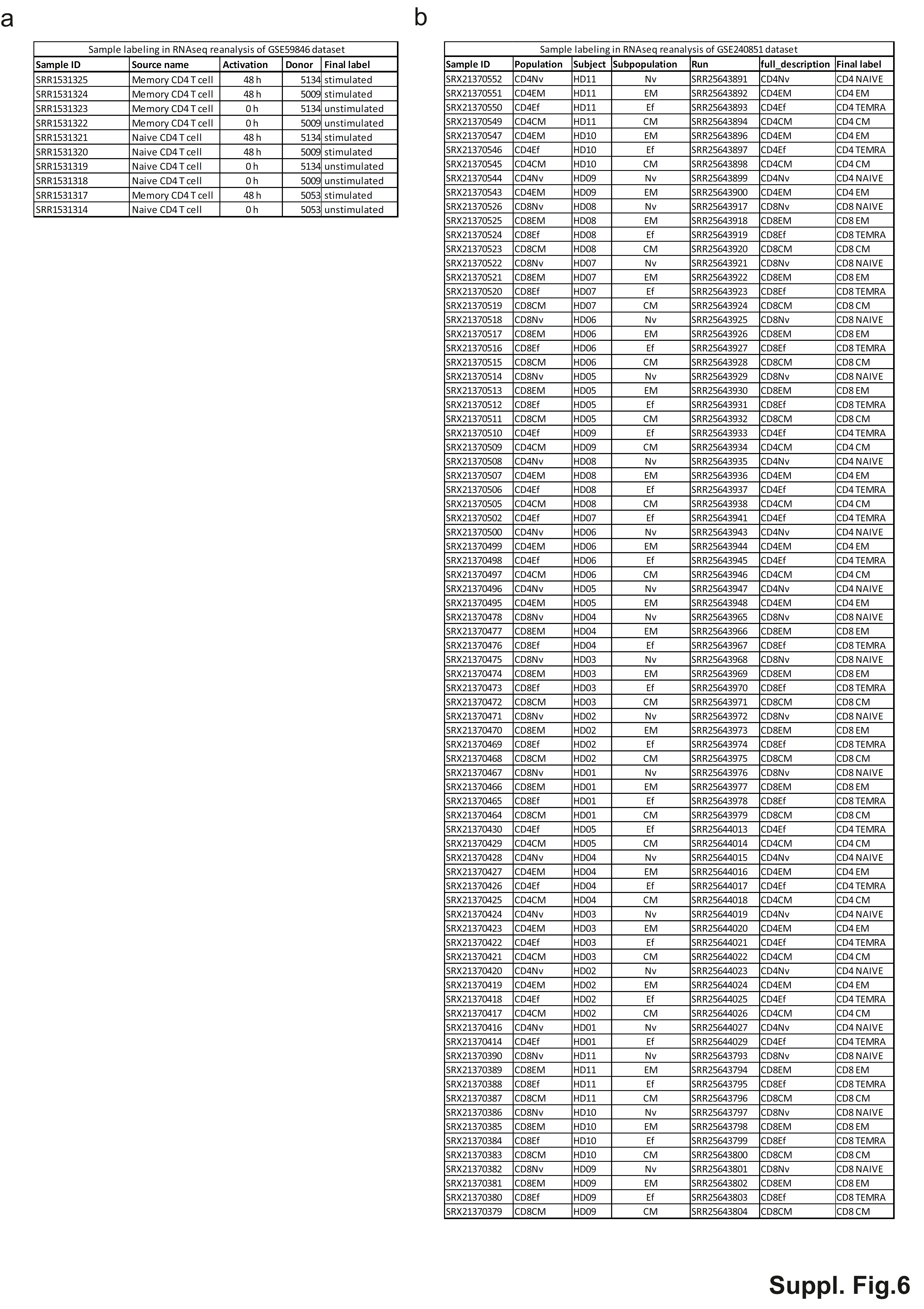


**Suppl. Fig. 6. Sample labeling in RNA-seq analysis.**

**a.** Table summarizing sample labeling information for the RNA-seq dataset GSE59846.
**b.** Table summarizing sample labeling information for the RNA-seq dataset GSE240851.


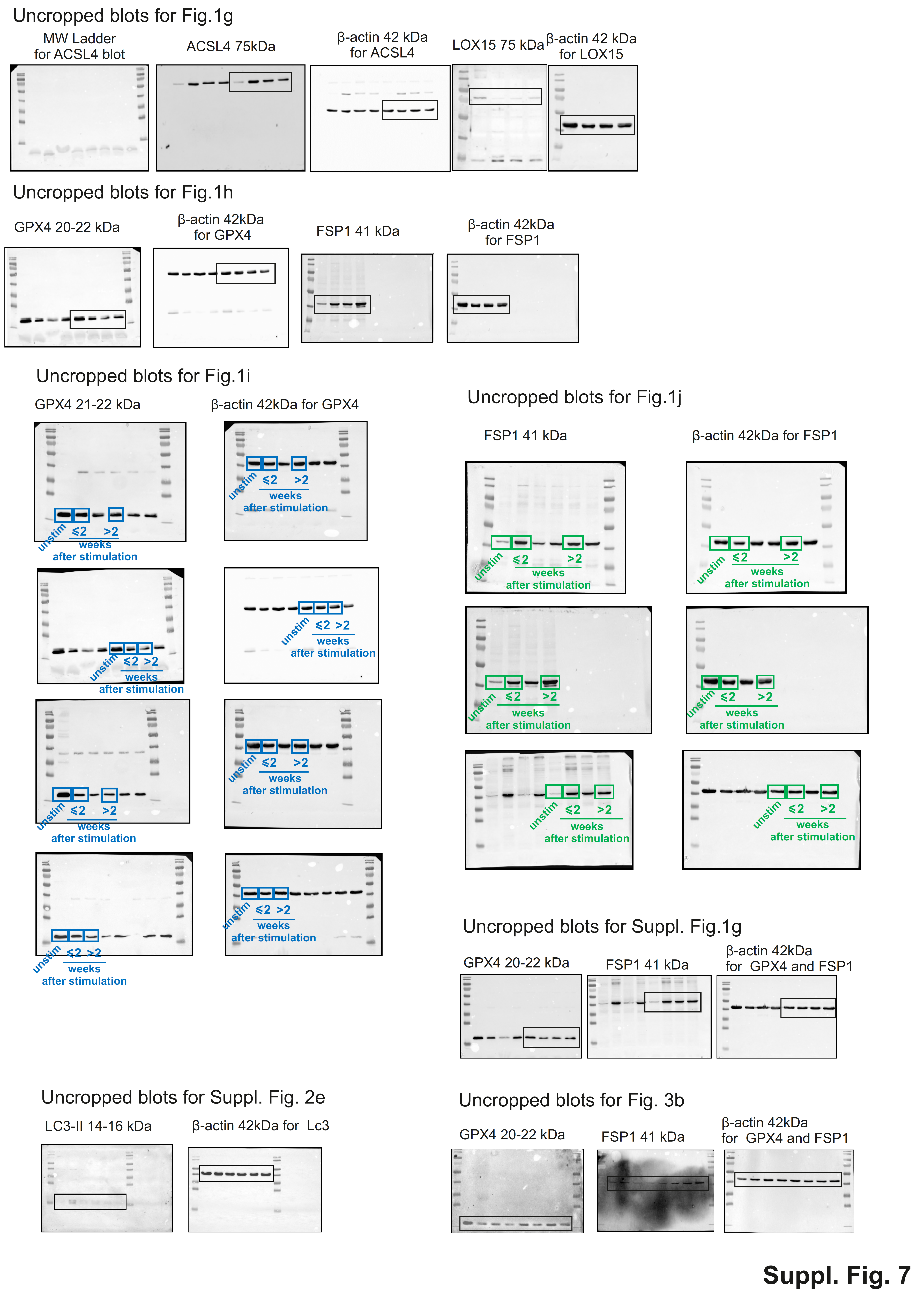


**Suppl. Fig. 7.** Uncropped blots corresponding to the Western blot results in the main figures. Protein bands corresponding to those in the main figures are highlighted with frames.
